# Integrated Epigenome Profiling of Repressive Histone Modifications, DNA Methylation and Gene Expression in Normal and Malignant Urothelial Cells

**DOI:** 10.1371/journal.pone.0032750

**Published:** 2012-03-07

**Authors:** Ewa Dudziec, Andreas Gogol-Döring, Victoria Cookson, Wei Chen, James Catto

**Affiliations:** 1 Institute for Cancer Studies and Academic Urology Unit, University of Sheffield, Sheffield, United Kingdom; 2 Max Delbrück Center for Molecular Medicine, Berlin, Germany; 3 Reproductive Medicine, University of Sheffield, Sheffield, United Kingdom; Roswell Park Cancer Institute, United States of America

## Abstract

Epigenetic regulation of gene expression is commonly altered in human cancer. We have observed alterations of DNA methylation and microRNA expression that reflect the biology of bladder cancer. This common disease arises by distinct pathways with low and high-grade differentiation. We hypothesized that epigenetic gene regulation reflects an interaction between histone and DNA modifications, and differences between normal and malignant urothelial cells represent carcinogenic events within bladder cancer. To test this we profiled two repressive histone modifications (H3K9m3 and H3K27m3) using ChIP-Seq, cytosine methylation using MeDIP and mRNA expression in normal and malignant urothelial cell lines. In genes with low expression we identified H3K27m3 and DNA methylation each in 20–30% of genes and both marks in 5% of genes. H3K9m3 was detected in 5–10% of genes but was not associated with overall expression. DNA methylation was more closely related to gene expression in malignant than normal cells. H3K27m3 was the epigenetic mark most specifically correlated to gene silencing. Our data suggest that urothelial carcinogenesis is accompanied by a loss of control of both DNA methylation and H3k27 methylation. From our observations we identified a panel of genes with cancer specific-epigenetic mediated aberrant expression including those with reported carcinogenic functions and members potentially mediating a positive epigenetic feedback loop. Pathway enrichment analysis revealed genes marked by H3K9m3 were involved with cell homeostasis, those marked by H3K27m3 mediated pro-carcinogenic processes and those marked with cytosine methylation were mixed in function. In 150 normal and malignant urothelial samples, our gene panel correctly estimated expression in 65% of its members. Hierarchical clustering revealed that this gene panel stratified samples according to the presence and phenotype of bladder cancer.

## Introduction

Bladder cancer is the fifth commonest malignancy in the United States with 70, 530 new cases and 14,680 deaths in 2010 [1]. The majority of tumors are Urothelial Cell Carcinoma (UCC). Clinicopathological data suggest this disease arises by two distinct pathways with low and high-grade cellular differentiation. The clinical phenotype and treatment of these two pathways differs considerably and molecular comparisons reveal few common events. The majority of UCC are low-grade tumors, which are characterized by FGFR3 mutation, chromosome 9 loss and relatively few other molecular alterations [2]. In contrast, high-grade tumors have widespread chromosomal instability, numerous molecular changes and are best characterized by loss of p53 function.

Molecular changes in cancer arise from either genetic or epigenetic events. The latter is defined as stable heritable changes in a chromosome without alterations in the DNA sequence [3]. Epigenetic gene modulation occurs when a stimulus, termed epigenator, induces a change in gene expression (e.g. by altered transcription or non-coding RNA) that becomes maintained within the genome through cell replication and in terminally differentiated cells [3] [4], [5]. Epigenetic maintainers induce an altered chromatin state by biochemical modification of DNA or histone proteins. Numerous histone modifications are described and these can be classified according location, biochemistry or associated gene expression. Of those that are repressive in nature, trimethylation (m3) of Histone 3 Lysine 9 (H3K9) and Histone 3 Lysine 27 (H3K27) are some of the best characterized [6], [7]. These epigenetic marks may occur independently or in combination with other modifications such as H3 lysine 4 methylation, H3K9 mono-methylation and H2A.Z [8]. At the nucleotide level DNA methylation mostly occurs at cytosine residues within CpG dinucleotides. These are concentrated into dense islands typically around the 5′ end of genes. Most human genes contain a CpG island and the majority of these are unmethylated to allow associated gene transcription [9]. Cytosine methylation may occur physiologically during development or aberrantly in carcinogenesis. Consequent tumor suppressor gene silencing or oncogene activation induces and promotes tumorogenesis. Whilst evidence suggests that epigenetic modifications of DNA and histone interact to modulate gene expression, the precise sequence and extent of this interaction is unclear and contrasting reports exist (reviewed in [10]).

We have previously observed changes in DNA methylation and microRNA expression that reflect the molecular biology of UCC and are associated with the clinical phenotype of tumors [5], [11], [12]. In particular, DNA methylation appears a common carcinogenic event that occurs early in the disease pathway [13] and an independent predictor of tumor progression [14]. Whilst indicating an important role for epigenetic gene regulation in UCC, these studies were limited to only one mechanistic tier of control and did not analyze histone alterations. To gain a more in depth knowledge of repressive epigenetic gene regulation in UCC, we have now profiled H3K9m3 and H3K27m3 in normal and malignant urothelial cells. We matched these profiles to those for 5-methylcytosine and gene expression. We hypothesized that differences represent pro-carcinogenic events within the urothelium.

## Results

### Histone enrichment in urothelial cells

**Figure 1:**
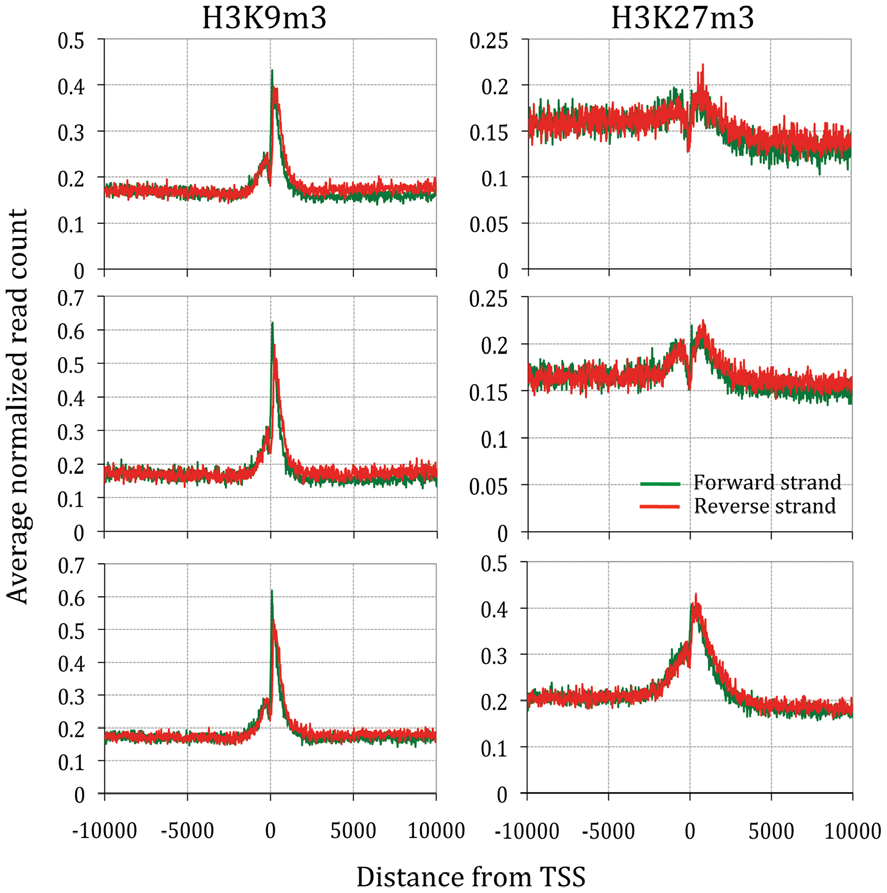
Enrichment for DNA bound to H3K9m3 and H3K27m3 around Transcriptions Start Sites (TSS). Although deep sequencing reads were mapped throughout the genome, there was enrichment around that varied between H3K9m3 and H3K27m3. The average number of reads within the 2 kb window surrounding 33,183 transcription start sites (TSS) is shown.

We performed massively parallel sequencing to determine the distribution of DNA adjacent to H3K9m3 and H3K27m3 in urothelial cells. Each experiment yielded between 7.7 M and 18.9 M reads (table S1, mean = 13.7 M s.d. = 4.4 M), of which 66–81% were mapped to unique genomic locations. Whilst reads were located throughout the entire genome, we observed enrichment around Transcription Start Sites (TSS, figure 1). The nature of TSS enrichment varied with histone modification [7]. For example, H3K9m3 enrichment was more specific to TSS than H3K27m3. When individual TSS were compared (n = 47,746, Figure S1a), few shared enrichment in all three cells (n = 151 for H3K9m3 and n = 277 for H3K27m3). When TSS with surrounding CpG islands were analyzed (n = 28,690), we observed a greater specificity for H3K9m3 (89–95% of TSS with enrichment had a CpG island) than for H3K27m3 (28–69%, T Test p<0.001, Figure S1b).

**Figure 2:**
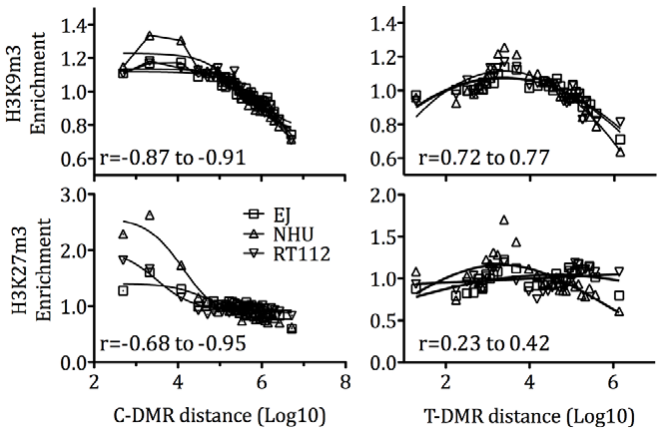
Histone enrichment according to the distance from Tissue and Cancer specific Differentially Methylated Regions (DMRs). We calculated the distance from gene TSS (n = 47,746) to the nearest DMR (C-DMR n = 2,708 and T-DMR n = 16,380) and binned genes into 30 sets (each dot represents one bin) for which we calculated average reads within a 10 kb window around the TSS for that bin. We normalized enrichment reads according to the total number for that experiment. For C-DMRs, a clear inverse correlation exists between TSS distance and histone enrichment (Pearson's correlation coefficients shown (r)). For T-DMRs the relationship appears more Gaussian in distribution.

Recent data suggest that many tissue and cancer-specific differentially DNA methylated regions (T and C-DMRs) occur within 2 kb around CpG islands (termed CpG island shores [15]). To examine the relationship between these DMRs and repressive histone modifications, we calculated enrichment within a 10 kb window around TSS according to the distance from the nearest C-DMR (n = 2,708) or T-DMR (n = 16,380) (figure 2). We observed a different relationship for each of the two classes of DMR. For C-DMRs, an inverse linear relationship between distance and enrichment fold was present for both histone modifications. This relationship was stronger for H3K9m3 and in cancer cell lines, when compared to H3K27m3 and NHU cells (Pearson coefficients r = −0.90 to −0.98 versus −0.77 to −0.83). Thus, TSS close to C-DMRs have higher enrichment for repressive histone modifications, than distant transcription sites. These data support our observation that H3K9m3 is more specific to TSS with CpG islands, than H3K27m3. The relationship between T-DMRs and histone modification was less clear. For H3K9m3 there appeared a Gaussian distribution with maximal read enrichment at 10^4^ bases distant. For H3K27m3, the relationship was bimodal with peak enrichment occurring at both 10^4^ and 10^7^ base distances.

### Histone enrichment and mRNA expression

**Figure 3:**
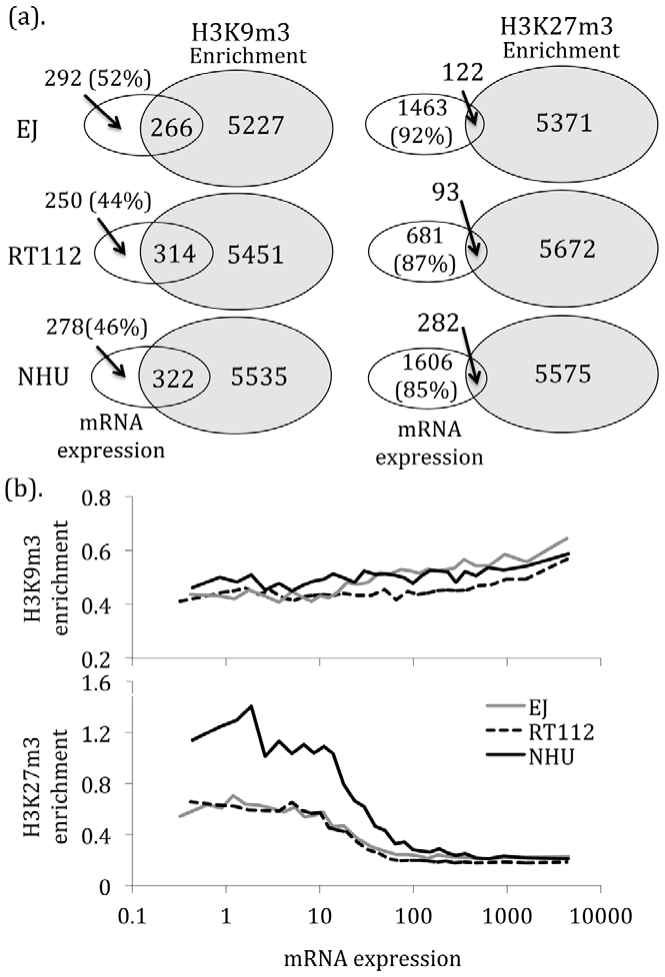
Histone enrichment and gene expression. We matched the histone enrichment to mRNA expression for 9,998 genes. All genes illustrated in these Venn diagrams were expressed, as determined by Microarray Analysis Suite 5 (see methods). (a). Quantitative analysis revealed that H3K27m3 enrichment was mostly mutually exclusive to gene expression. In contrast, up to 52% of TSS with H3K9m3 enrichment had gene expression. (b). Qualitative analysis reveals that H3K27m3 enrichment is inversely correlated with mRNA expression, in contrast to H3K9m3. This plot demonstrates average histone enrichment for the 9,998 genes when grouped into 30 bins according to mRNA expression.

To compare histone modifications with gene expression, we combined our sequencing data with mRNA expression. Due to differences in enrichment profiles, used sequencing reads from a 5 kb window around the TSS for H3K9m3 and from the entire gene for H3K27m3. Matching data were available for 9,998 genes. H3K27m3 was mostly associated with gene silencing. In total, 85–92% of expressed genes did not have H3K27m3 enrichment (figure 3a) and quantified gene expression appeared inversely correlated with enrichment (r = −0.93 to −0.97, p<0.0001, figure 3b). TSS with H3K27m3 enrichment had a less than 10 fold expression than those without this mark (ANOVA p<0.0001, Figure S2). In contrast, mRNA expression was often seen with H3K9m3 enrichment (48–56% of enriched genes had mRNA expression). Indeed, enrichment and mRNA expression appeared positively correlated when quantified (r = 0.64 to 0.9, p<0.0001, figure 3b).

### DNA methylation, mRNA expression and histone enrichment

**Figure 4:**
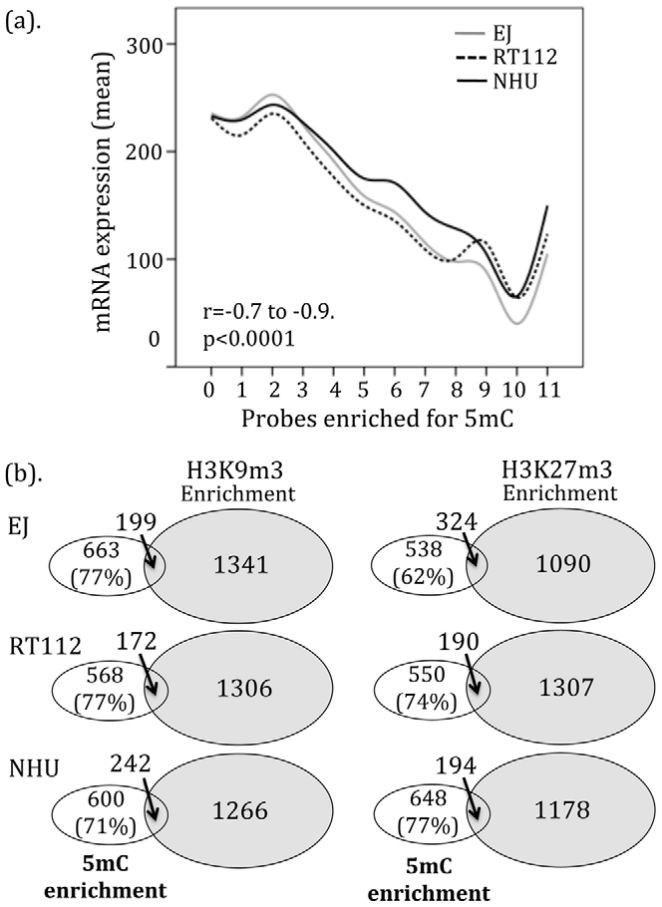
Cytosine methylation and gene expression. (a). Gene expression was inverse to the density of adjacent probes enriched for 5 mC (data from 10,568 genes (corresponding to 9,799 CpG islands)). (b). When compared, methylation of 5 mC and H3K9m3 or H3K27m3 appeared usually mutually exclusive. Of the 6809 genes with matching data, less than 5% shared methylation of 5 mC and either histone residue.

Using MeDIP and CpG island microarrays we identified DNA regions with and without 5 mC enrichment. We selected the 20% most enriched probes (defined as DNA hypermethylation), filtered for those with differences between the malignant and normal cells (n = 86,414) and selected probes with differential hypermethylation in the cancerous lines (n = 68,292 (79%)). We mapped these probes to their nearest neighbors to identify regions with adjacent (those <200 bp apart) enriched probes. Depending upon stringency, we found between 1,513 (5/5 adjacent probes) and 106 (11/11 adjacent probes) CpG islands around 711 and 40 protein-coding genes, respectively. Of these, 20% and 26% were located within a promoter and the remainder within (66% and 8%) or downstream from the nearest gene. We annotated the 5 mC profile in each cell line for these 68,292 probes using mRNA expression and obtained data for 10,568 genes (corresponding to 9,799 CpG islands). When measured an inverse correlation was seen between mRNA expression and 5 mC enrichment for these probes (r = −0.7 to −0.9, p<0.001, figure 4). Reduced gene expression appeared proportional to the extent of 5 mC enrichment. We combined the 5 mC and histone enrichments data to obtain matching patterns for 6,809 genes (figure 4b). Between 23 and 38% of genes had both epigenetic modifications. In contrast to mRNA expression, there appeared no differences between H3K9m3 and H3K27m3 with respect to cell lines or 5 mC enrichment.

### Multilayered epigenetic repression of gene expression

**Figure 5:**
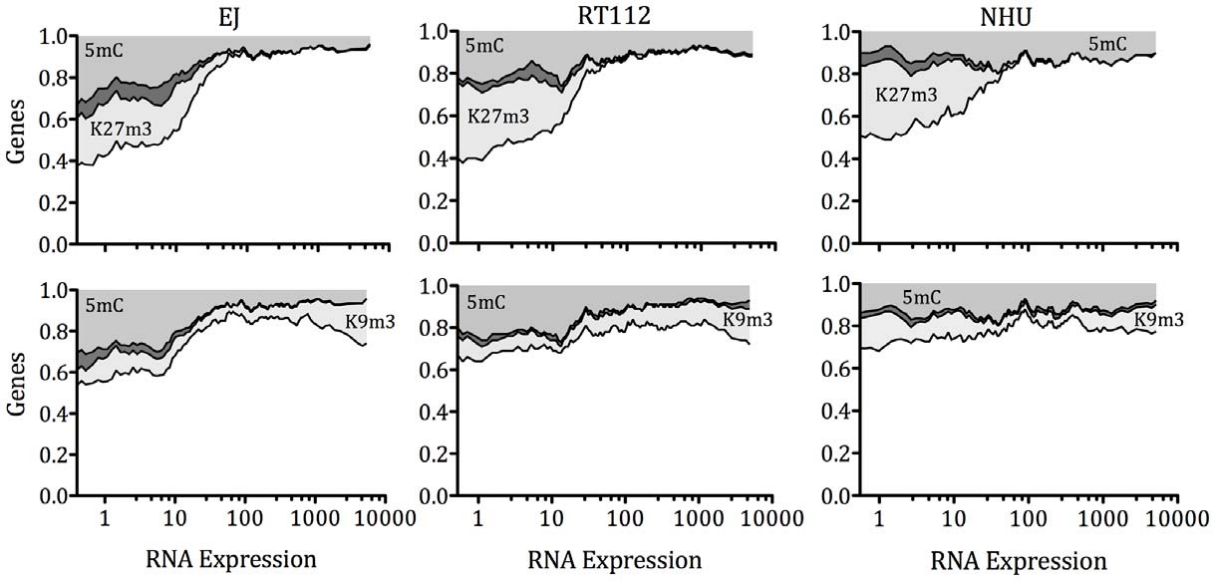
Integrated epigenetic gene expression. For each cell line, we have plotted the proportion of genes with enrichment for 5 mC (light grey area), H3K9m3 or H3K27m3 (light grey area), or both (dark grey area) with respect to gene expression. We calculated this plot for 6809 genes using sliding windows (each covering 5% of all loci) at 1% intervals. H3K27m3 enrichment is limited to genes with low expression. H3K9m3 and 5 mC enrichment persist for genes with high expression. Few loci share epigenetic marks.

To profile epigenetic events in gene repression, we combined histone enrichment, DNA hypermethylation and gene expression datasets (matching data for 6,809 genes). In general, DNA and histone enrichment were negatively correlated to mRNA expression (figure 5). This relationship appeared most specific for H3K27m3, as many genes with high expression had enrichment for H3K9m3. For example, at low mRNA expression levels in EJ 26–28% of genes were enriched for H3K27m3, 11–17% had associated DNA hypermethylation and 4–6% shared both traits. The proportion of genes with these epigenetic marks reduced to less than 5% as mRNA expression increased. The inverse relationship between H3K9m3 and gene expression was less strong and direct than for H3K27m3. At low mRNA expression, 11–16% of genes were enriched for H3K9m3, 16–21% had associated DNA hypermethylation and 2–3% shared both traits. Of interest, the pattern of enrichment for epigenetic marks did not vary between malignant and normal cell lines.

To determine specific loci important for urothelial carcinogenesis we identified those with cancer-specific silencing and associated epigenetic modifications. Specifically, we selected genes with one or more repressive epigenetic mark (defined as within the highest 20% of enrichment) that was shared in the two cancer lines and absent in NHU. From these, we chose genes with reduced expression in EJ and/or RT112, and preserved expression in NHU. This strategy identified 239 and 317 genes in EJ and RT112, respectively (Figure S3a). Of these, 88 genes had low expression in both cancer lines. H3K27m3 and DNA hypermethylation appeared the commonest epigenetic traits (Figure S3b). Alone or in combination these events were found in 49–58% and 54–59% of silenced genes, respectively. Only 6–7% of silenced genes had isolated H3K9m3 enrichment. Shared patterns of enrichment for H3K9m3 or H3K27m3, and DNA hypermethylation were present in the two cell lines for 8(9%), 34(39%) and 29(33%) of these 88 genes, respectively.

### Multilayered epigenetic gene upregulation

To evaluate epigenetically mediated gene upregulation in cancer we used the combined dataset (n = 6,809 genes) to identify those with DNA hypomethylation and/or loss of enrichment for either histone modification in the cancer cell lines, when compared to NHU. From these we selected genes with cancer specific expression (i.e. were silenced in NHU cells, Figure S4). In total, we found 355 and 373 genes in EJ and RT112, respectively. Of these, 114 genes were silenced in NHU and present in both cancer lines. DNA hypomethylation appeared the most common epigenetic trait and was present in for 54–73% of these genes. Reduced relative H3K27m3 and H3K9m3 enrichment were found in 28–33% and 4–12% of genes, respectively.

### External validation of genes with carcinogenic aberrant epigenetic regulation

We combined the epigenetic silencing and upregulation gene panels to create a list encompassing tumor specific changes within our cells (n = 202, table S2). To explore this panel we firstly performed pathway analysis using gene enrichment software [16] and illustrated using area-proportional Venn diagrams [17]. Whilst the cohort enriched for 5 mC was largest, gene enrichment analyses suggested that those with associated H3K27m3 were most biologically active (110 gene clusters, Figure S5, table S3). Pathway annotation revealed that genes marked with H3K27m3 represented mitosis/cell division/chromatin assembly (enrichment 2.12, p = 0.005), regulation of transcription (1.63, p = 0.009), protein phosphorylation (1.38, p = 0.01), induction of apoptosis and cell migration/cytoskeleton organization (1.1, p = 0.03). For H3K9m3 there were fewest gene clusters and these represented members of the response to nutrient/extracellular stimulus (1.1, p = 0.01). Genes with only 5 mC enrichment were clustered into pathways dealing with the response to stress/oxidation (1.8, p = 0.002), cell morphogenesis and movement. Finally, genes with all three epigenetic marks were concentrated into 5 clusters involving members of the nucleoside and nucleotide binding, regulation of gene expression and transcription pathways. Differences also occurred between genes with cancer associated epigenetic silencing and upregulation. Whilst the former group was smaller (88 genes) its members were clustered into 71 pathways, in contrast to the 47 for the upregulated (114 gene) panel.

**Figure 6:**
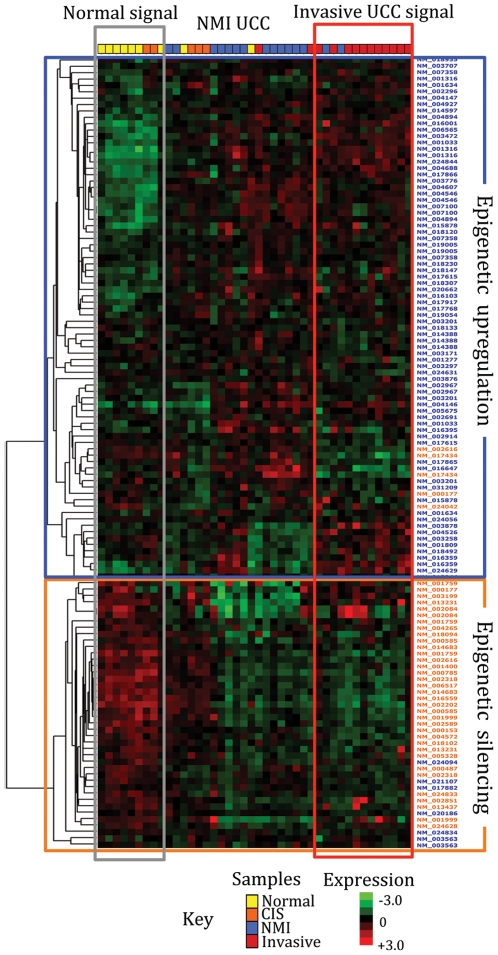
Clustering of urothelial samples using genes with epigenetic alteration in UCC cell lines. Unsupervised hierarchical clustering using genes filtered to those present within our epigenetic gene panel stratified urothelial samples according to the presence and phenotype of cancer. Previously reported microarray data [18] was filtered for members of our epigenetic panel (blue: upregulated, orange: down regulated) whose expression was correctly predicted. Unsupervised average linkage hierarchical clustering was performed on median centered genes using Cluster 3.0. The selected genes stratify normal and malignant samples mostly according to phenotype (CIS: Carcinoma in situ, NMI: Non-muscle invasive UCC). For simplicity only normal urothelium and primary UCC were used in the analysis.

To examine the extent of aberrant expression for members of our epigenetic panel in human UCC we interrogated two microarray datasets generated using a related Affymetrix platform (U133A, n = 22,283 probesets (n = 13,703 genes) [18]) and the largest published with UCC [19]. In the former, we identified 136 gene members from our silenced (58/88) and upregulated (78/114) panels, and compared their expression in the 9 normal urothelial and 46 UCC samples. In total, 95/136 (70%) of genes had their differential expression correctly predicted by our epigenetic marks (ANOVA p<0.05, Figure S6). Unsupervised hierarchical clustering separated the samples according to their phenotype (figure 6), suggesting that our cell line panel reflects events within primary human cancers. In the second dataset, we identified 177 members of our panel and correctly predicted the expression of 106 (60%). Once again unsupervised hierarchical clustering using our panel members stratified 10 normal and 85 malignant samples according to phenotype (Figure S7). We determined expression of our identified epigenetic panel members in each tumor sub-type (table S4), as low and high grade UCC arise by distinct tumor pathways. For each tumor phenotype we saw common and specific events. For example, there were 28 genes specific to invasive tumors when compared to non-muscle invasive disease and 67 that appeared common to UCC.

## Discussion

Here we have produced integrated epigenomic maps for two UCC cell lines and non-transformed normal urothelial cells. The latter are cultured expansions taken from disease free patients that grow as sheets of histologically normal appearing urothelial cells for 7–8 passages before senescence. Our experimental data reveal insights into the epigenetic control of gene expression in the biology of UCC and have specifically identified genes potentially involved in urothelial carcinogenesis.

At the global level several observations are apparent. Firstly, H3K9m3 and H3K27m3 are located mostly around gene transcription start sites [7]. In the UCC lines we observed that H3K27m3 was less specifically bound to TSS and more evenly distributed throughout the gene, than for NHU. This may reflect alterations in H3K27 methylation in UCC, perhaps due to changes in EZH2 or UTX expression. The EZH2 polycomb protein specifically methylates H3K27 and is commonly overexpressed in UCC [20]. Indeed our gene expression data revealed EZH2 mRNA was upregulated in both cells when compared to NHU (data not shown)). UTX is a H3K27m3 demethylase. A recent report has described a hemizygous deletion (through mutation) in RT112 cells [21], suggesting deficiency in the removal of methyl residues in this line. Secondly, our genome wide sequencing identified few TSS that shared epigenetic marks between cell lines (1.3–3.7% of mapped TSS). This may reflect differences in cell phenotype and supports previous observations defining the diversity of histone modifications [8]. Wang et al. profiled 37 different histone acetylation and methylation modifications in CD4+ cells and detected 4,339 different combinations (of which 3,165 were at only one gene site). Only 1,018 of the 12,541 genes studied had H3K27me3 enrichment (in keeping with our observations).

Our data detail the associations between H3K9m3, H3K27m3, CpG islands and DMRs (mostly CpG shores [15]). Of the histone modifications, H3K9m3 appeared most specific to CpG regions and was almost exclusively bound to TSS with CpG islands (89–96%, Figure S1). Consequently enrichment for H3K9m3 was inversely correlated to distance from CpG shores (figure 2). The specificity of H3K9m3 for CpG islands supports an interaction with cytosine methylation [10], although these epigenetic modifications coexisted infrequently in our cells. Our analysis suggests that the relationship between 5 mC and H3K9m3 is strongest in genes with absent or low expression, but does persist into those with high expression. This lack of specificity suggests that neither event is sufficient for gene silencing and may explain the low proportion of genes with both epigenetic marks in our cells. In contrast, H3K27m3 was less specifically located to TSS with CpG islands (28–69%) and consequently less correlated to DMR distance than H3K9m3. Whilst overall there was little relationship between H3K27m3 and cytosine methylation, a direct correlation existed in genes with low expression. Whilst this association may be because both marks are negatively correlated to gene expression, it could reflect a direct causation (as they affect distinct TSS). Whilst our methodology has identified associations between epigenetic marks and gene expression, we did not examine this cause or direction. Data to examine this relationship may be obtained from the literature. Schlesinger et al. identified that genes silenced in cancer are initially associated with H3K27m3 [22]. This mark is maintained by EZH2, which recruits DNA methyl transferases and these in turn methylate previously unmethylated cytosine residues. Rush et al. then identified that EZH2 specifically recruits DNMT3a, but that this alone is insufficient for de novo methylation, suggesting a need for additional events [23]. Support for this order of events could be found in silenced genes with H3K27m3 but not DNA methylation. We identified many such examples, as did Kondo et al. [24].

The clearest observations from our data are those regarding the integrated nature of epigenetic gene regulation in the urothelium. In malignant cells, at low gene expression around 30% of loci had associated 5 mC, around 20–30% had associated H3K27m3 enrichment and 5% had both epigenetic marks (figure 5). Genes with both epigenetic marks included potentially carcinogenic members such as BCL11A, EHD3 and HAS2 (table S2) and drive key malignant pathways such as apoptosis avoidance and gene transcription (table S3). For normal urothelial cells, at low gene expression a similar proportion of loci had H3K27m3 (30%) enrichment, but only 15% had associated cytosine methylation. When the epigenetic marks are analyzed with respect to gene expression, H3K27m3 appears a specific mark of low gene expression, 5 mC appears a partial mark of low expression in malignant cells and H3K9m3 does not appear to be related to gene expression. In normal cells, 5 mC did not appear to be strongly correlated with gene expression. These are important findings, that suggest the dynamic nature of epigenetic modifications and reveal the importance of H3K27m3 in epigenetic gene repression. When taken with the observations that EZH2 maintains H3K27m3 and recruits DNA methyl transferases, our data suggest that the transition from normal to malignant urothelium is accompanied by a loss of control of both DNA methylation and H3k27 methylation. When analyzed in detail, we found alterations of these two epigenetic events occurred in 1/4 of genes (table S2).

Our analyses identified 202 genes whose epigenetic marks and differential gene expression suggested an involvement in urothelial carcinogenesis. For the majority of these genes the epigenetic event was either H3K27m3 or 5 mC. Gene enrichment pathway analysis suggested diverse roles for the three epigenetic marks. For example, genes marked by H3K9m3 were involved in cellular metabolism and the response to external stimuli. These important cellular pathways need to remain constant within a cell and not vary with transformation. In contrast, genes marked with H3K27m3 appeared carcinogenic in function and were involved with cell division, chromatin assembly, regulation of transcription, the induction of apoptosis and cell migration. Genes with only 5 mC enrichment were clustered into pathways dealing with the response to stress and oxidation, cell morphogenesis and movement. These pathways represent a mixture of those important for cell homeostasis and those involved in cancer. Given our findings (that 5 mC varies with urothelial carcinogenesis), one would suspect that the pro-carcinogenic pathways associated with 5 mC are those contributing to malignant transformation.

To assess the validity of the genes identified using cell lines, we examined their expression in 150 normal and malignant urothelial samples. Our epigenetic panel correctly estimated the expression of 65% of genes and stratified the tissues according to the presence and phenotype of cancer. This is important as previous epigenetic studies using candidate tumor suppressor genes reveal that low-grade non-invasive cancers differ to high grade and invasive UCC [11], [12]. Our identified gene panel includes many members with suspected roles in carcinogenic pathways. For example, FEZ1/LZTS1 (Leucine zipper putative tumour suppressor 1) is a tumour suppressor gene important for cell cycle control. FEZ1 is located at chromosome 8p22 in a region deleted in many cancers, including 42% of UCC [25]. Whilst loss of FEZ1 expression has been identified in UCC [26], the mechanism has remained unclear [25]. Our data now suggest that epigenetic repression in association with H3K27m3 is responsible. Gelsolin (GSN) is involved in actin filament assembly and disassembly. Loss of GSN expression is detected in UCC and produces changes in cytoskeletal structure typical for cancer [27]. Our data suggest H3K27m3 mediated repression is responsible for GSN silencing and support observations of GSN upregulation when cells are exposed to histone deacetylase inhibition [28].

One of the most interesting candidates within our panel is the Cellular Apoptosis Susceptibility/CSE1 chromosome segregation 1-like gene (hCAS/CSE1L). This component of the nuclear import pathway is frequently upregulated in cancer and located at 20q13 within a region amplified in malignancy [29]. The carcinogenic role for CSE1L was first detected in apoptosis avoidance and recently it has been identified as a mediator of p53 function and to associate with chromatin [30]. Specifically, CSE1L decreased H3K27 methylation at certain p53 target genes to enable their expression. That we have observed upregulation of CSE1L in association with reduced H3K27m3 suggests a potential positive feedback loop whereby increased CSE1L expression facilitates further CSE1L upregulation.

In conclusion, we have mapped repressive epigenetic events in malignant and normal urothelial cells. In genes with low expression we identified associated H3K27m3 and DNA methylation each in 20–30% of genes and both marks in 5% of genes. When all genes were analyzed H3K9m3 did not appear to be associated with expression. DNA methylation was more closely related to gene expression in cancerous than normal cells. H3K27m3 was the epigenetic mark most specifically correlated to gene silencing. We identified a panel of genes with cancer specific epigenetic mediated aberrant expression including those with known carcinogenic functions and members potentially mediating a positive epigenetic feedback loop.

## Materials and Methods

### Cell lines and nucleic acid extraction

We analyzed bladder cancer cell lines representing non-invasive and invasive disease (RT112 and EJ/T24, purchased from ATCC) grown in Dulbecco's medium with 10% fetal calf serum, and normal human urothelial (NHU) cells maintained in keratinocyte serum-free medium containing bovine pituitary extract, epidermal growth factor (Invitrogen, Paisley, UK) and cholera toxin [31]. NHU cells are non-immortalized and derived from histologically confirmed normal urothelium obtained from patients without a history of UCC, using standard methods. From each cell line we extracted DNA using the QIAquick Purification Kit (QIAGEN, UK) and RNA using the mirVana™ kit (Ambion, TX), according to manufacturer's specifications (methods detailed elsewhere [12], [32]). Nucleic acid concentrations were measured using a 2100 Bioanalyzer (Agilent, Cheshire, UK).

### Chromatin Immunoprecipitation and Solexa sequencing (ChIP-Seq)

Malignant and normal human urothelial cells were cross-linked with 1% formaldehyde in PBS for 10 min at room temperature. Glycine was added to the final concentration of 0.125 M and the mixture incubated for 5 min to quench unreacted formaldehyde. Cross-linked cells were washed with PBS, lysed in 250 µl SDS lysis buffer and sonicated (Bioruptor 200, Diagenode, Liège, Belgium). Samples were pre-cleared with 15 µl of protein G dynabeads (Invitrogen) and 50 µl supernatant saved as total input control. Rotating samples were immunoprecipitated overnight at 4°C with antibodies against H3K9m3 (Millipore, Watford, UK), H3K27m3 (Millipore) and rabbit IgG as a negative control (Santa Cruz Biotechnology, CA). The chromatin:antibody complex was incubated with 20 µl dynabeads protein G for 1 h at 4°C, before washing and DNA elution. Cross-links were reverted by overnight incubation with RNAse A at 65°C before DNA purification using columns (Qiagen, Germany). Immunoprecipitated DNA ends were repaired using the PNK and Klenow enzyme, adenosine was added to the 3′ end of the fragments and the DNA was ligated with the adapters. Following ligation, the ChIP DNA was amplified for 17 cycles and fragments around 200–300 bp isolated using electrophoresis in an agarose gel. The purified DNA was used for cluster generation and sequencing analysis using the Solexa 1 G Genome Analyzer according to manufacturer protocols (Illumina, CA) as detailed elsewhere [33]. The sequencing reads (36 bp) were mapped using Bowtie [34] to the chromosomes of the human genome (NCBI built GRCh37/hg19, February 2009) allowing up to two mismatches (command line argument –v 2). Only uniquely mapped reads were used for the subsequent analysis. If several reads were mapped to the same position and strand, we counted them as a single read.

### Genome wide profiling of DNA methylation

Methylated DNA immunoprecipitation and tiling CpG island microarrays (Human CpG Island Microarray, Agilent, CA) (MeDIP-CHIP) were used to determine genome wide distribution of 5 mC [35]. Briefly, genomic DNA was sheared by sonication to yield 200–600 bp fragments and incubated with antibodies raised to either 5 mC (anti-5-methylcytidine, Eurogentec, Hampshire, UK) or murine IgG (negative control). The antibody-antigen complex was captured with magnetic beads conjugated to anti-mouse-IgG (Santa Cruz Biotechnology) and washed, unbound, non-specific DNA removed, before methylated DNA elution. Successful enrichment was determined using quantified PCR for EDNRB (known to be methylated in these cells [14]]). The immunoprecipitated (Cyanine 5-dUTP) and reference DNA (Cyanine 3-dUTP) were then labeled (Genomic DNA Enzymatic Labeling Kit, Agilent), cleaned (Amicon filters, Millipore) and quantified. Competitive hybridization onto the microarray was then performed (ChIP-on-Chip Hybridization Kit, Agilent) according to manufacturer's instructions in a rotating SureHyb chamber at 67°C for 40 hours. Washed slides were scanned (High-Resolution C Scanner, Agilent) and fluorescence obtained using Feature Extraction software. The microarray contains 244,000 probes that tile through 27,800 CpG features at an average of 100 bp separation. To calculate methylation, we averaged probes within each CpG region and defined those within the highest and lowest quintiles (20%) of relative Cy5 fluorescence as hyper or hypo-methylated, respectively.

### mRNA expression

Whole genome mRNA expression was determined by microarray (HG-U133 Plus 2.0, Affymetrix, Cal.) as detailed elsewhere [36]. This platform contains 54,000 probesets, including 33,000 to known coding genes. Briefly, RNA was prepared using the Affymetrix protocol (enzymes from Invitrogen) and annealed to an oligo-d(T) primer with a T7 polymerase binding site. cDNA was generated using superscript II and E. coli DNA ligase and polymerase I. The reaction was completed with T4 DNA polymerase and EDTA. Amplified cDNA was cleaned, biotin-labeled, fragmented and hybridized to the microarray for 16 hours at 45°C in a rotating oven at 60 rpm. After washing and staining, the arrays were scanned (GC3000 scanner) and data processed using Gene Chip Operating System software. mRNA expression was determined using Microarray Analysis Suite 5 (Affymetrix) and defined as expressed (perfect match probeset intensity greater than mismatch intensity) or absent (mismatch probeset intensity greater or equal to perfect match intensity).

### External validation and statistical analysis

Our analyses identified a panel of genes that appeared to be important in urothelial carcinogenesis. To evaluate their role in human UCC, we extracted expression data from Array Express (www. generated using related Affymetrix microarrays [18] and the largest published within this tumor [19]. We analyzed the selected genes using unsupervised hierarchical clustering with Cluster 3.0 and TreeView (Eisen lab) as described [12]. For statistical comparisons we compared epigenetic events using a Chi squared test for discrete variables and ANOVA for continuous data. All statistical analyses were two sided and performed in SPSS (Vsn. 14.0 SPSS Inc, ILL) using p<0.05 for significance.

### Data deposition

The raw data from each experiment is deposited on line at NCBI GEO with accession numbers GSE 31125, GSE 31864, GSE 31865 and GSE 31866.

## Supporting Information

Figure S1 **Histone enrichment at individual Transcription Start Sites.** The number of TSS with shared enrichment for between each of the three cell lines is shown for (a). all genes and (b). for genes around CpG islands. As can been seen, H3K9m3 appeared more specific to CpG islands than H3K27m3.
(PDF)

Figure S2 **Gene expression and histone enrichment.** Gene expression was an average of more than 10 fold lower in TSS with H3K27m3 enrichment, compared to those without enrichment (ANOVA p<0.0001, figure 3b). For H3K9m3 little difference in gene expression was seen, with respect to enrichment.
(PDF)

Figure S3 **Epigenetic gene silencing in bladder cancer.** Venn diagrams represent number of down regulated genes in EJ and RT112 bladder cancer cell lines, when compared to NHU, associated with each epigenetic mark.
(PDF)

Figure S4 **Epigenetic gene upregulation in bladder cancer.** Venn diagrams represent number of upregulated genes in EJ and RT112 bladder cancer cell lines, when compared to NHU, associated with each epigenetic mark.
(PDF)

Figure S5 **Functional annotation clustering of genes with epigenetic marks in EJ cells.** Using gene enrichment pathway analysis we determined clusters of genes for each set marked by an epigenetic modification. The number of gene clusters within each part of the diagram is indicated in these area proportional Venn diagrams.
(PDF)

Figure S6 **The expression of genes within our combined epigenetic panel in an external dataset** [**18**] **.** The median expression of 124 genes is shown when stratified according to the epigenetic traits found in EJ, RT112 and NHU. As shown, genes with predicted epigenetic upregulation had higher expression than those with predicted silencing.
(PDF)

Figure S7 **Unsupervised hierarchical clustering stratified malignant and normal urothelial samples according to phenotype.** Previously reported microarray data [19] was filtered for members of our epigenetic panel (blue: upregulated, orange: downregulated) whose expression was correctly predicted. Unsupervised average linkage hierarchical clustering was performed on median centered genes using Cluster 3.0. The selected genes stratify normal and malignant samples mostly according to phenotype (Yellow: Normal, Blue: Non-muscle invasive and Red: Invasive/metastatic). For simplicity we evaluated only normal urothelium from control patients without cancer, and tumors characteristic of the low grade and invasive pathways.
(PDF)

Table S1 **Reads per experiment obtained from massively parallel sequencing.**
(PDF)

Table S2 **Details of the combined epigenetic gene panel stratified for associated events and mRNA expression.** Below each column is the proportion of total events (as a percentage).
(PDF)

Table S3 **Gene ontology analysis for each epigenetic mark.** The table reveals the GO details for all identified pathways associated with each event.
(PDF)

Table S4 **Phenotype specific gene expression in the epigenetically selected panel as determined using gene expression data** [**17**] **.**
(PDF)

## References

[1] Jemal A, Siegel R, Xu J, Ward E Cancer statistics (2010). CA Cancer.. J Clin.

[2] van Oers JM, Zwarthoff EC, Rehman I, Azzouzi AR, Cussenot O (2009). FGFR3 mutations indicate better survival in invasive upper urinary tract and bladder tumours.. Eur Urol.

[3] Berger SL, Kouzarides T, Shiekhattar R, Shilatifard A (2009). An operational definition of epigenetics.. Genes & development.

[4] Catto JW, Alcaraz A, Bjartell AS, De Vere White R, Evans CP (2011). MicroRNA in Prostate, Bladder, and Kidney Cancer: A Systematic Review.. Eur Urol.

[5] Dudziec E, Miah S, Choudhry HM, Owen HC, Blizard S (2011). Hypermethylation of CpG islands and shores around specific microRNAs and mirtrons is associated with the phenotype and presence of bladder cancer.. Clin Cancer Res.

[6] Kouzarides T (2007). Chromatin modifications and their function.. Cell.

[7] Barski A, Cuddapah S, Cui K, Roh TY, Schones DE (2007). High-resolution profiling of histone methylations in the human genome.. Cell.

[8] Wang Z, Zang C, Rosenfeld JA, Schones DE, Barski A (2008). Combinatorial patterns of histone acetylations and methylations in the human genome.. Nat Genetics.

[9] Sharma S, Kelly TK, Jones PA (2010). Epigenetics in cancer.. Carcinogenesis.

[10] Fuks F (2005). DNA methylation and histone modifications: teaming up to silence genes.. Current Opinion Genetics & Dev.

[11] Catto JW, Azzouzi AR, Rehman I, Feeley KM, Cross SS (2005). Promoter hypermethylation is associated with tumor location, stage, and subsequent progression in transitional cell carcinoma.. J Clin Oncol.

[12] Catto JW, Miah S, Owen HC, Bryant H, Dudziec E (2009). Distinct microRNA alterations characterize high and low grade bladder cancer.. Cancer Res.

[13] Dhawan D, Hamdy FC, Rehman I, Patterson J, Cross SS (2006). Evidence for the early onset of aberrant promoter methylation in urothelial carcinoma.. J Pathol.

[14] Yates DR, Rehman I, Abbod MF, Meuth M, Cross SS (2007). Promoter hypermethylation identifies progression risk in bladder cancer.. Clin Cancer Res.

[15] Irizarry RA, Ladd-Acosta C, Wen B, Wu Z, Montano C (2009). The human colon cancer methylome shows similar hypo- and hypermethylation at conserved tissue-specific CpG island shores.. Nat Genetics.

[16] Huang da W, Sherman BT, Lempicki RA (2009). Systematic and integrative analysis of large gene lists using DAVID bioinformatics resources.. Nat Protoc.

[17] Hulsen T, de Vlieg J, Alkema W (2008). BioVenn - a web application for the comparison and visualization of biological lists using area-proportional Venn diagrams.. BMC genomics.

[18] Dyrskjot L, Kruhoffer M, Thykjaer T, Marcussen N, Jensen JL (2004). Gene expression in the urinary bladder: a common carcinoma in situ gene expression signature exists disregarding histopathological classification.. Cancer Res.

[19] Kim WJ, Kim EJ, Kim SK, Kim YJ, Ha YS (2010). Predictive value of progression-related gene classifier in primary non-muscle invasive bladder cancer.. Mol Cancer.

[20] Raman JD, Mongan NP, Tickoo SK, Boorjian SA, Scherr DS (2005). Increased expression of the polycomb group gene, EZH2, in transitional cell carcinoma of the bladder.. Clin Cancer Res.

[21] van Haaften G, Dalgliesh GL, Davies H, Chen L, Bignell G (2009). Somatic mutations of the histone H3K27 demethylase gene UTX in human cancer.. Nat Genetics.

[22] Schlesinger Y, Straussman R, Keshet I, Farkash S, Hecht M (2007). Polycomb-mediated methylation on Lys27 of histone H3 pre-marks genes for de novo methylation in cancer.. Nat Genetics.

[23] Rush M, Appanah R, Lee S, Lam LL, Goyal P (2009). Targeting of EZH2 to a defined genomic site is sufficient for recruitment of Dnmt3a but not de novo DNA methylation.. Epigenetics.

[24] Kondo Y, Shen L, Cheng AS, Ahmed S, Boumber Y (2008). Gene silencing in cancer by histone H3 lysine 27 trimethylation independent of promoter DNA methylation.. Nat Genetics.

[25] Knowles MA, Aveyard JS, Taylor CF, Harnden P, Bass S (2005). Mutation analysis of the 8p candidate tumour suppressor genes DBC2 (RHOBTB2) and LZTS1 in bladder cancer.. Cancer Letters.

[26] Ishii H, Baffa R, Numata SI, Murakumo Y, Rattan S (1999). The FEZ1 gene at chromosome 8p22 encodes a leucine-zipper protein, and its expression is altered in multiple human tumors.. Proc Natl Acad Sci USA.

[27] Tanaka M, Mullauer L, Ogiso Y, Fujita H, Moriya S (1995). Gelsolin: a candidate for suppressor of human bladder cancer.. Cancer Res.

[28] Gould JJ, Kenney PA, Rieger-Christ KM, Silva Neto B, Wszolek MF (2010). Identification of tumor and invasion suppressor gene modulators in bladder cancer by different classes of histone deacetylase inhibitors using reverse phase protein arrays.. J Urol.

[29] Behrens P, Brinkmann U, Wellmann A (2003). CSE1L/CAS: its role in proliferation and apoptosis.. Apoptosis.

[30] Tanaka T, Ohkubo S, Tatsuno I, Prives C (2007). hCAS/CSE1L associates with chromatin and regulates expression of select p53 target genes.. Cell.

[31] Southgate J, Hutton KA, Thomas DF, Trejdosiewicz LK (1994). Normal human urothelial cells in vitro: proliferation and induction of stratification.. Lab Invest.

[32] Catto JW, Azzouzi AR, Amira N, Rehman I, Feeley KM (2003). Distinct patterns of microsatellite instability are seen in tumours of the urinary tract.. Oncogene.

[33] Adamidi C, Wang Y, Gruen D, Mastrobuoni G, You X (2011). De novo assembly and validation of planaria transcriptome by massive parallel sequencing and shotgun proteomics.. Genome Res.

[34] Langmead B (2010). Aligning short sequencing reads with Bowtie.. Current protocols in bioinformatics/editoral board, Andreas D Baxevanis [et al] Chapter.

[35] Mohn F, Weber M, Schubeler D, Roloff TC (2009). Methylated DNA immunoprecipitation (MeDIP).. Methods Mol Biol.

[36] Ferraiuolo L, Heath PR, Holden H, Kasher P, Kirby J (2007). Microarray analysis of the cellular pathways involved in the adaptation to and progression of motor neuron injury in the SOD1 G93A mouse model of familial ALS.. J Neurosci.

