# Supplementary material for: Integrated Epigenome Profiling of Repressive Histone Modifications, DNA Methylation and Gene Expression in Normal and Malignant Urothelial Cells
Source: PLoS One. 2012 Mar 7;7(3):e32750. doi: 10.1371/journal.pone.0032750 (PMC3296741; doi:10.1371/journal.pone.0032750)
Supplement: Figure S1 — Histone enrichment at individual Transcription Start Sites. The number of TSS with shared enrichment for between each of the three cell lines is shown for (a). all genes and (b). for genes around CpG islands. As can been seen, H3K9m3 appeared more specific to CpG islands than H3K27m3. (PDF) [file pone.0032750.s001.pdf]

a). Enrichment at all individual TSS (n=47,746)

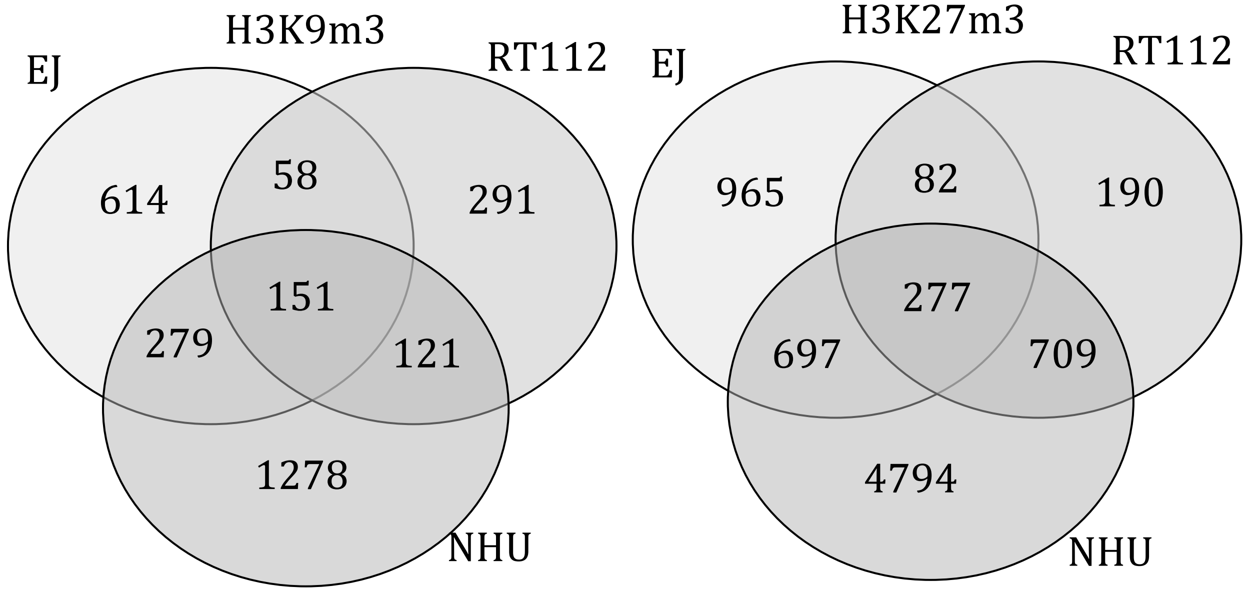

b). Enrichment of DNA at individual TSS with CpG islands (n=28,690). The percentage of total TSS is shown.

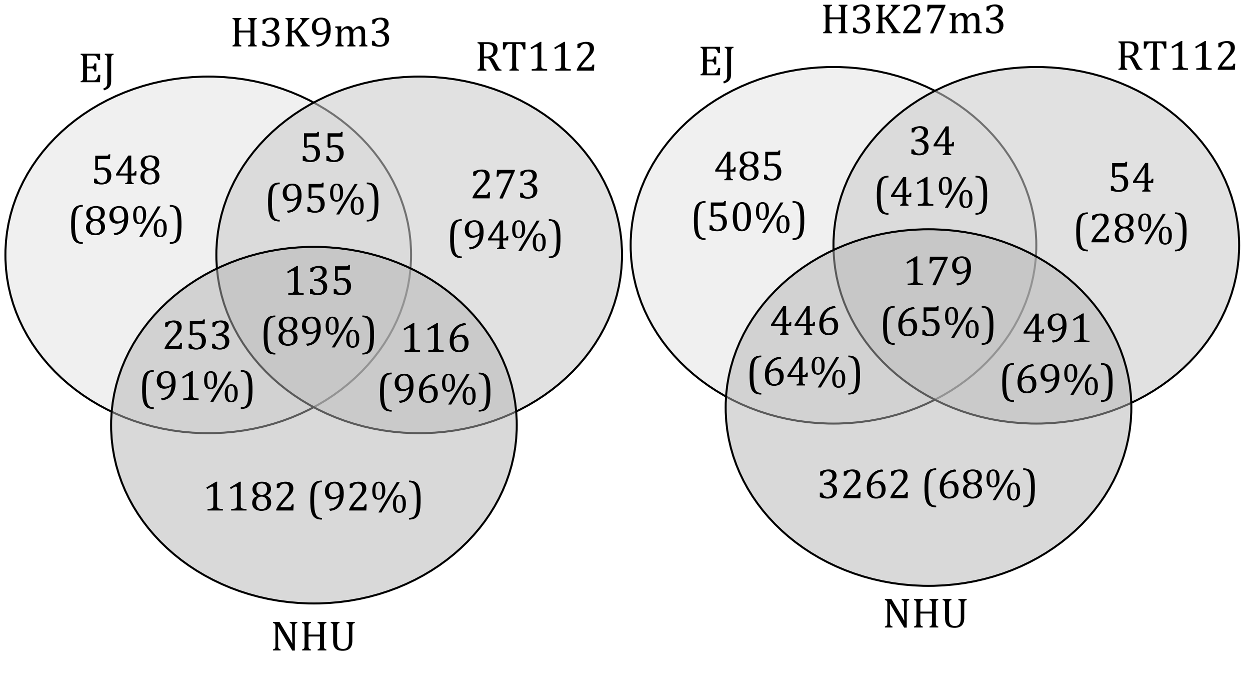

Supplementary figure 1: Histone enrichment at specific TSS
