# Supplementary figures and images for: Integrated Epigenome Profiling of Repressive Histone Modifications, DNA Methylation and Gene Expression in Normal and Malignant Urothelial Cells
Source: PLoS One. 2012 Mar 7;7(3):e32750. doi: 10.1371/journal.pone.0032750 (PMC3296741; doi:10.1371/journal.pone.0032750)

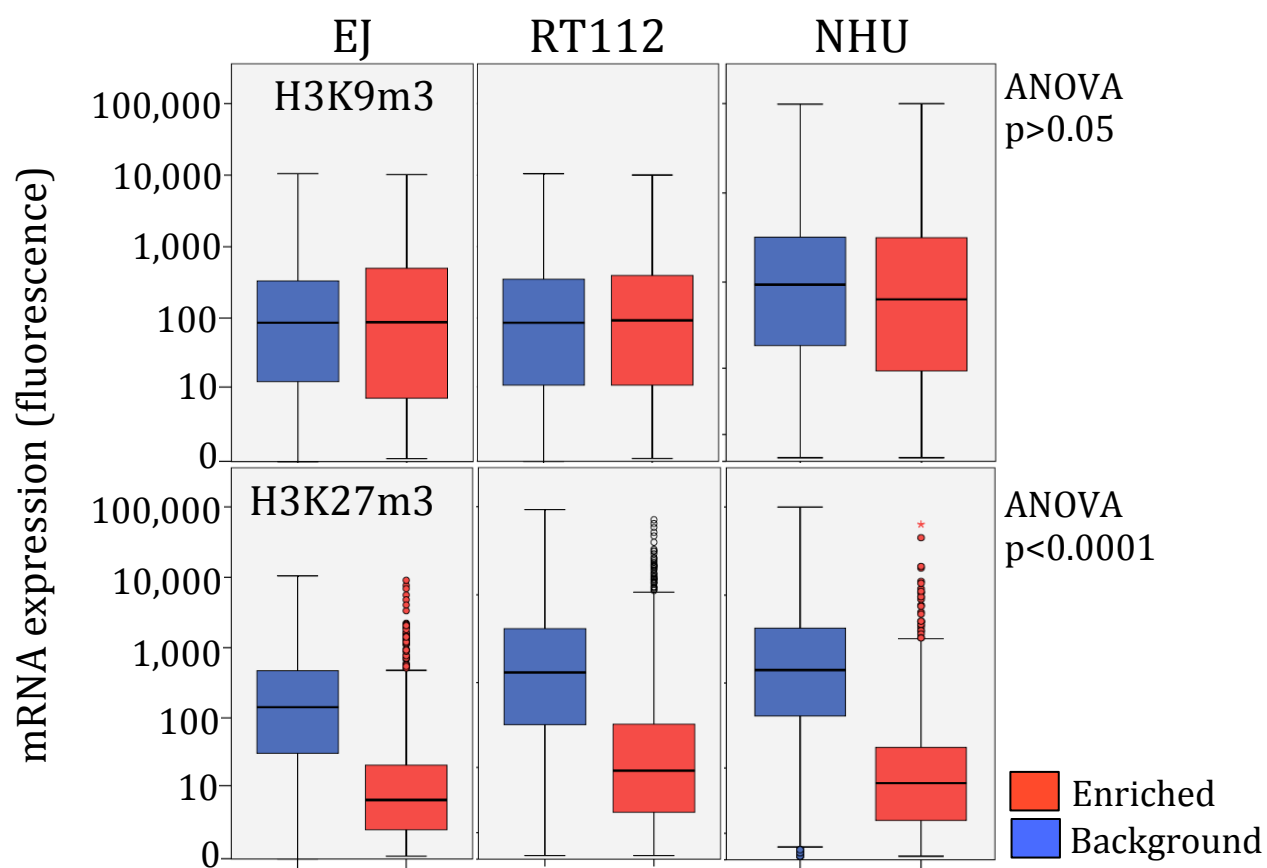

Supplementary figure 2: Gene expression and histone enrichment.

Supplement: Figure S2 — Gene expression and histone enrichment. Gene expression was an average of more than 10 fold lower in TSS with H3K27m3 enrichment, compared to those without enrichment (ANOVA p<0.0001, figure 3b). For H3K9m3 little difference in gene expression was seen, with respect to enrichment. (PDF) [file pone.0032750.s002.pdf]
