# Supplementary material for: Integrated Epigenome Profiling of Repressive Histone Modifications, DNA Methylation and Gene Expression in Normal and Malignant Urothelial Cells
Source: PLoS One. 2012 Mar 7;7(3):e32750. doi: 10.1371/journal.pone.0032750 (PMC3296741; doi:10.1371/journal.pone.0032750)
Supplement: Figure S4 — Epigenetic gene upregulation in bladder cancer. Venn diagrams represent number of upregulated genes in EJ and RT112 bladder cancer cell lines, when compared to NHU, associated with each epigenetic mark. (PDF) [file pone.0032750.s004.pdf]

a). Cell lines analyzed in isolation

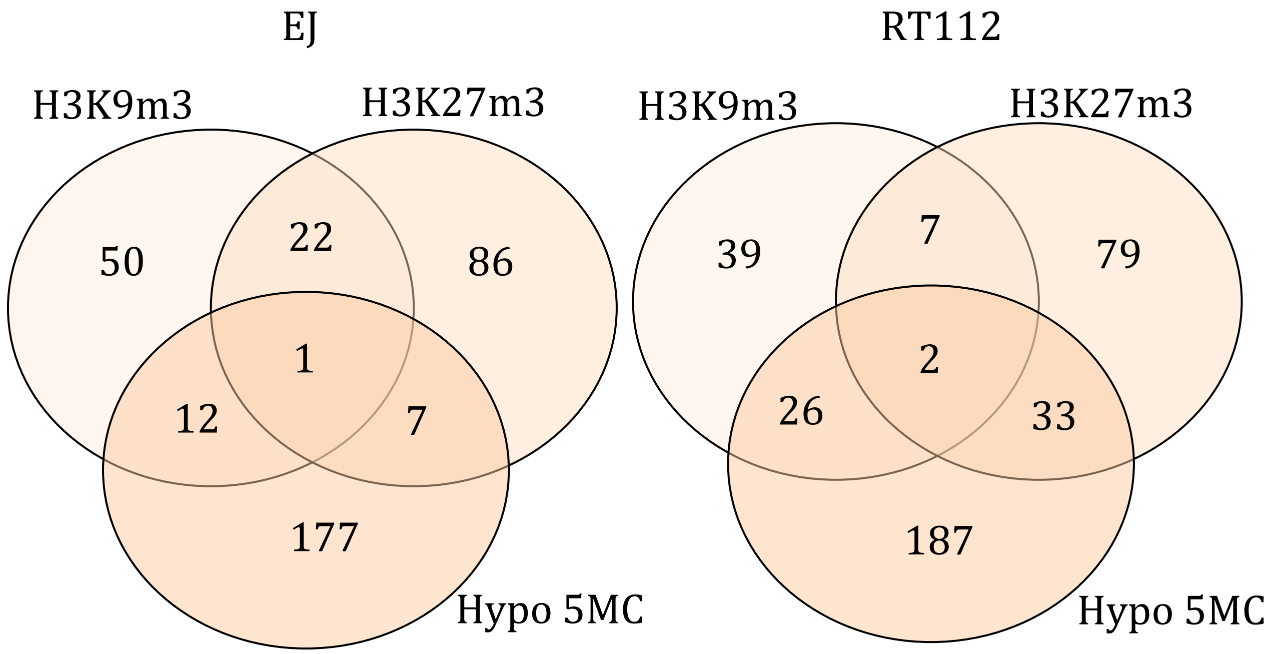

b). Genes common to both cancer cell lines

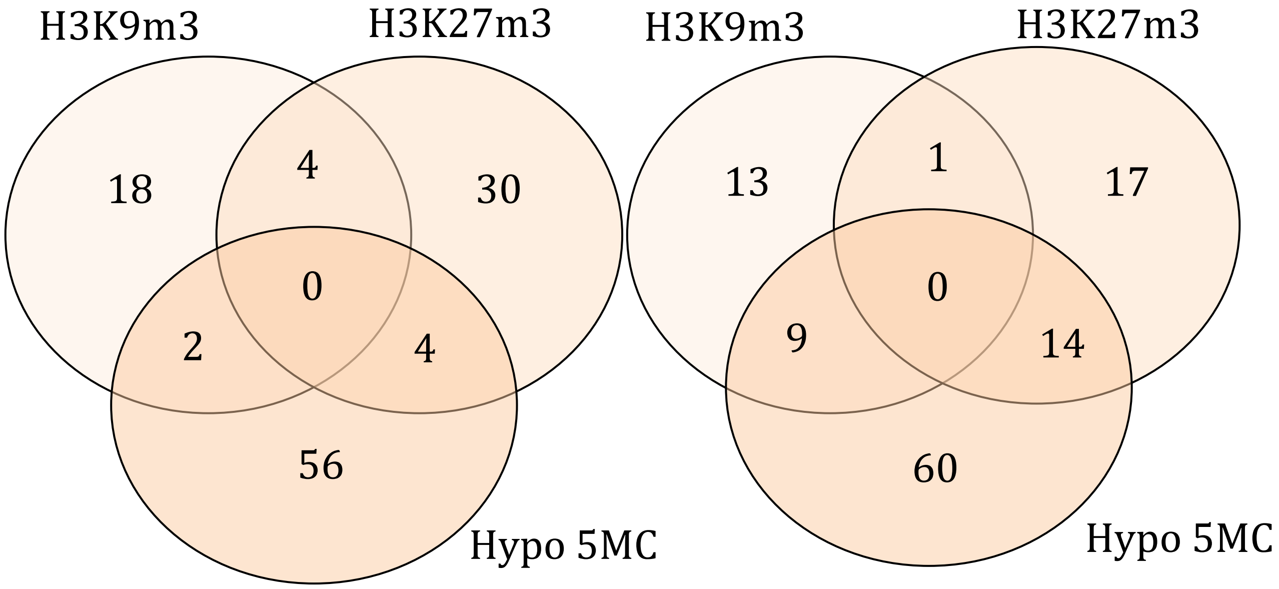

Supplementary figure 4: Epigenetic gene upregulation in malignant cells when compared to NHU
