# Supplementary material for: Integrated Epigenome Profiling of Repressive Histone Modifications, DNA Methylation and Gene Expression in Normal and Malignant Urothelial Cells
Source: PLoS One. 2012 Mar 7;7(3):e32750. doi: 10.1371/journal.pone.0032750 (PMC3296741; doi:10.1371/journal.pone.0032750)
Supplement: Figure S5 — Functional annotation clustering of genes with epigenetic marks in EJ cells. Using gene enrichment pathway analysis we determined clusters of genes for each set marked by an epigenetic modification. The number of gene clusters within each part of the diagram is indicated in these area proportional Venn diagrams. (PDF) [file pone.0032750.s005.pdf]

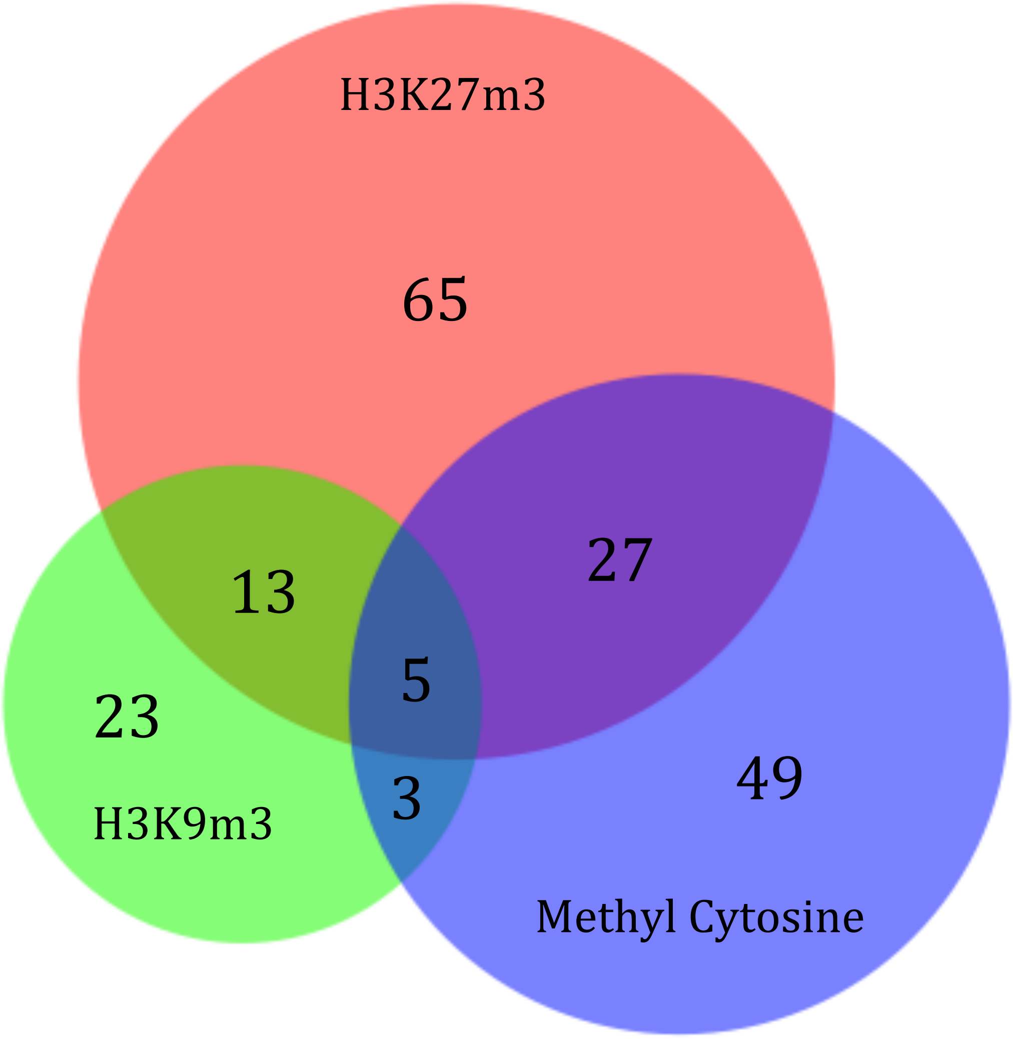

Supplementary figure 5: Functional annotation clustering of genes with epigenetic marks in EJ cells. The number of gene clusters within each part of the diagram is indicated.
