# Supplementary material for: Integrated Epigenome Profiling of Repressive Histone Modifications, DNA Methylation and Gene Expression in Normal and Malignant Urothelial Cells
Source: PLoS One. 2012 Mar 7;7(3):e32750. doi: 10.1371/journal.pone.0032750 (PMC3296741; doi:10.1371/journal.pone.0032750)
Supplement: Figure S6 — The expression of genes within our combined epigenetic panel in an external dataset [18] . The median expression of 124 genes is shown when stratified according to the epigenetic traits found in EJ, RT112 and NHU. As shown, genes with predicted epigenetic upregulation had higher expression than those with predicted silencing. (PDF) [file pone.0032750.s006.pdf]

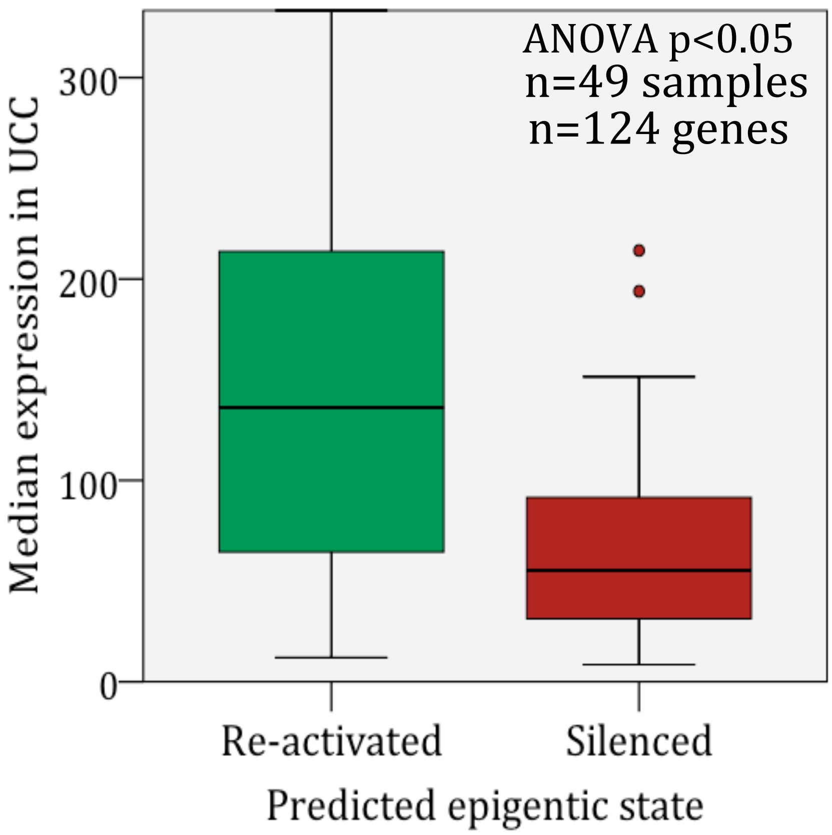

Supplementary figure 6: mRNA expression of our selected genes in 55 urothelial samples was lower in genes with repressive epigentic marks when compared to those predicted to be re-activated.
