# Supplementary material for: Integrated Epigenome Profiling of Repressive Histone Modifications, DNA Methylation and Gene Expression in Normal and Malignant Urothelial Cells
Source: PLoS One. 2012 Mar 7;7(3):e32750. doi: 10.1371/journal.pone.0032750 (PMC3296741; doi:10.1371/journal.pone.0032750)
Supplement: Figure S7 — Unsupervised hierarchical clustering stratified malignant and normal urothelial samples according to phenotype. Previously reported microarray data [19] was filtered for members of our epigenetic panel (blue: upregulated, orange: downregulated) whose expression was correctly predicted. Unsupervised average linkage hierarchical clustering was performed on median centered genes using Cluster 3.0. The selected genes stratify normal and malignant samples mostly according to phenotype (Yellow: Normal, Blue: Non-muscle invasive and Red: Invasive/metastatic). For simplicity we evaluated only normal urothelium from control patients without cancer, and tumors characteristic of the low grade and invasive pathways. (PDF) [file pone.0032750.s007.pdf]

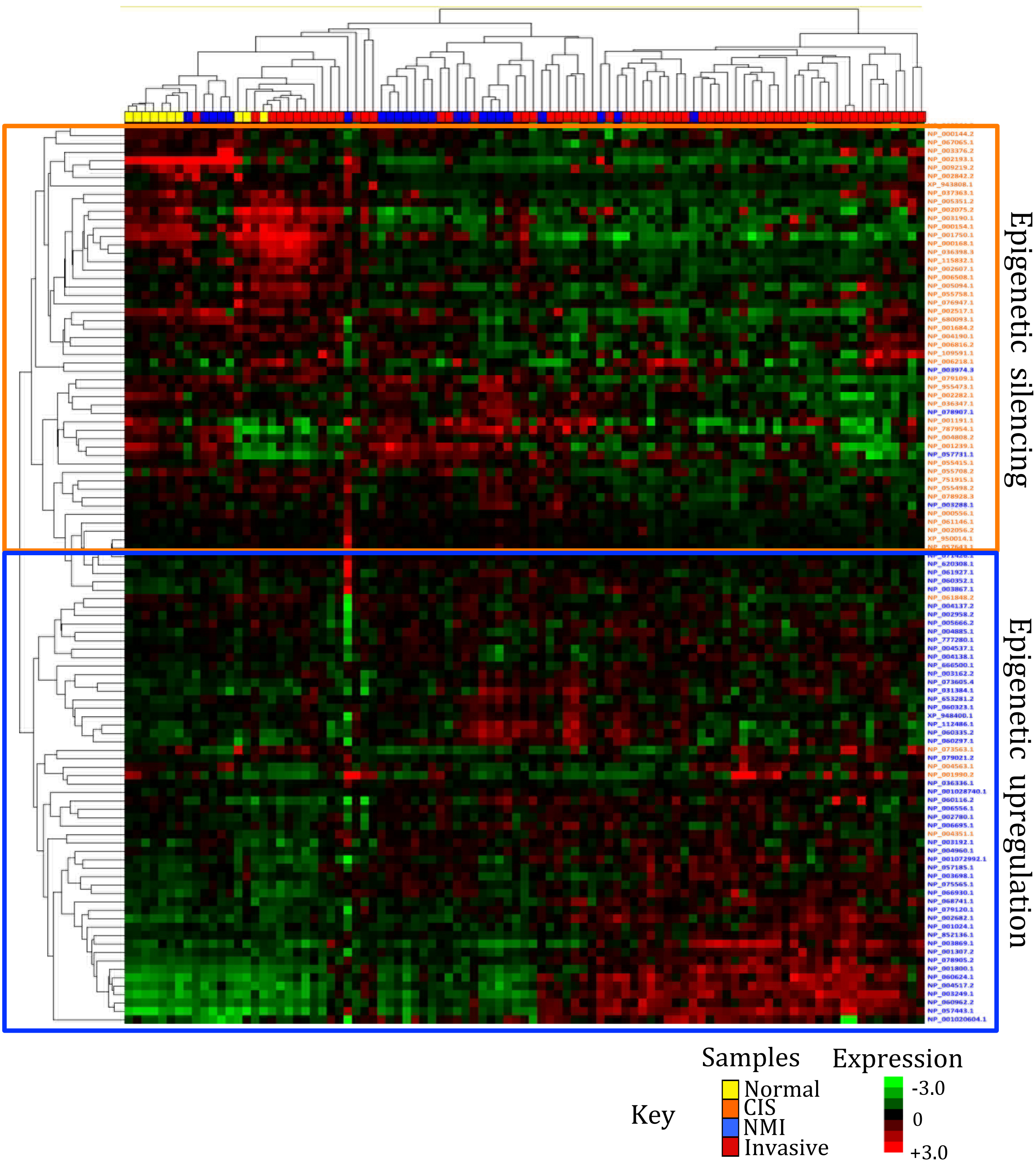

Supplementary figure 7. Unsupervised hierarchical clustering stratified malignant and normal urothelial samples according to phenotype
