# Supplementary material for: Integrated Epigenome Profiling of Repressive Histone Modifications, DNA Methylation and Gene Expression in Normal and Malignant Urothelial Cells
Source: PLoS One. 2012 Mar 7;7(3):e32750. doi: 10.1371/journal.pone.0032750 (PMC3296741; doi:10.1371/journal.pone.0032750)
Supplement: Table S1 — Reads per experiment obtained from massively parallel sequencing. (PDF) [file pone.0032750.s008.pdf]

Supplementary Table 1. Reads per experiment obtained from massively parallel sequencing.

Datasets	Raw reads	Mapped reads		Uniquely mapped reads	
H3K9m3 EJ	18,759,874	14,400,543	90.80%	10,500,982	66.21%
H3K27m3 EJ	18,908,844	14,875,620	97.23%	12,401,536	81.06%
H3K9m3 RTII2	11,386,627	9,738,322	96.88%	7,977,658	79.37%
H3K27m3 RTII2	7,649,773	6,352,143	92.80%	4,512,004	65.92%
H3K9m3 NHU	12,983,653	10,487,562	92.41%	7,678,788	67.66%
H3K27m3 NHU	12,490,239	10,635,276	96.62%	8,674,436	78.81%
