# Supplementary material for: Integrated Epigenome Profiling of Repressive Histone Modifications, DNA Methylation and Gene Expression in Normal and Malignant Urothelial Cells
Source: PLoS One. 2012 Mar 7;7(3):e32750. doi: 10.1371/journal.pone.0032750 (PMC3296741; doi:10.1371/journal.pone.0032750)
Supplement: Table S2 — Details of the combined epigenetic gene panel stratified for associated events and mRNA expression. Below each column is the proportion of total events (as a percentage). (PDF) [file pone.0032750.s009.pdf]

Supplementary table 2. Details of the combined epigenetic gene panel stratified for epigenetic mediated silencing and upregulation.

Silencing

		RNA		H3K27-3M			H3K9-3M			DNA 5mC		
Gene	Symbol	EJ-NHU	RT112-NHU	EJ	RT112	NHU	EJ	RT112	NHU	EJ	RT112	NHU
NM_265	GLUL	-1	-1	1			1	1		1	1	
NM_24833	ZNF671	-1	-1		1		1	1		1	1	
NM_1759	CCND2	-1	-1	1	1		1	1				
NM_785	CYP27B1	-1	-1	1	1		1				1	
NM_1999	FBN2	-1	-1				1	1		1	1	
NM_13231	FLRT2	-1	-1	1	1		1			1		
NM_15478	L3MBTL1	-1	-1	1	1		1				1	
NM_633	LIPG	-1	-1	1	1		1				1	
NM_2462	MX1	-1	-1	1	1			1			1	
NM_2589	PCDH7	-1	-1	1			1	1			1	
NM_1693	ATP6V1B2	-1	-1	1	1					1		
NM_1814	BCL11A	-1	-1	1	1					1		
NM_16229	CYB5R2	-1	-1	1						1	1	
NM_64	DCTN2	-1	-1	1	1						1	
NM_146	EHD3	-1	-1	1	1					1		
NM_22726	ELOVL4	-1	-1	1	1						1	
NM_4265	FADS2	-1	-1		1					1	1	
NM_14164	FXYD5	-1	-1					1		1	1	
NM_153	GALC	-1	-1	1						1	1	
NM_163	GHR	-1	-1	1	1					1		
NM_1894	GSPT2	-1	-1	1	1					1		
NM_5328	HAS2	-1	-1	1	1					1		
NM_565	IL6R	-1	-1	1	1						1	
NM_222	ISL1	-1	-1		1					1	1	
NM_2291	LAMB1	-1	-1	1			1	1				
NM_3915	LBH	-1	-1	1	1					1		
NM_13437	LRP12	-1	-1		1			1		1		
NM_24652	LRRK1	-1	-1	1			1				1	
NM_536	MAF	-1	-1	1	1					1		
NM_2442	METRN	-1	-1	1						1	1	
NM_17459	MFAP2	-1	-1	1						1	1	
NM_21242	MID1IP1	-1	-1	1				1			1	
NM_2526	NT5E	-1	-1		1		1			1		
NM_4199	P4HA2	-1	-1	1			1				1	
NM_445	PLEC	-1	-1	1				1			1	
NM_6227	PLTP	-1	-1				1			1	1	
NM_6822	RAB4B	-1	-1	1	1						1	
NM_14	S1PR1	-1	-1	1	1					1		
NM_24628	SLC12A8	-1	-1		1					1	1	
NM_6517	SLC16A2	-1	-1		1			1		1		
NM_18676	THSD1	-1	-1	1	1						1	
NM_1812	ZNF334	-1	-1	1	1		1					
NM_3816	ADAM9	-1	-1							1	1	
NM_487	ARSA	-1	-1				1	1				
NM_4874	BAG4	-1	-1							1	1	
NM_5881	BCKDK	-1	-1							1	1	
NM_12	BMP2	-1	-1	1	1							
NM_153218	C13orf31	-1	-1	1							1	
NM_436	CDH1	-1	-1							1	1	
NM_6825	CKAP4	-1	-1							1	1	
NM_198	CTSB	-1	-1							1	1	
NM_12266	DNAJB5	-1	-1	1	1							
NM_17434	DUOX1	-1	-1							1	1	
NM_24712	ELMO3	-1	-1							1	1	
NM_1248	ENTPD3	-1	-1	1	1							
NM_22749	FAM16B2	-1	-1							1	1	
NM_513	FEZ1	-1	-1	1	1							
NM_635	FST	-1	-1	1	1							
NM_284	GPX3	-1	-1							1	1	
NM_177	GSN	-1	-1	1							1	
NM_853	GSTT1	-1	-1							1	1	
NM_585	IL15	-1	-1							1	1	
NM_418	IL4R	-1	-1				1	1				
NM_5569	LIMK2	-1	-1				1				1	
NM_2318	LOXL2	-1	-1							1	1	
NM_61	MANF	-1	-1							1	1	
NM_12215	MGEA5	-1	-1				1				1	
NM_7287	MME	-1	-1	1	1							
NM_14893	NLGN4Y	-1	-1							1	1	
NM_2581	NYNRIN	-1	-1	1							1	
NM_2616	PER1	-1	-1				1				1	
NM_16559	PEX5L	-1	-1		1					1		
NM_4572	PKP2	-1	-1	1							1	
NM_5167	PPM1J	-1	-1	1							1	
NM_2851	PTPRZ1	-1	-1	1	1							
NM_16321	RHCG	-1	-1							1	1	
NM_3666	SERPINB1	-1	-1	1	1							
NM_18418	SPATA7	-1	-1	1	1							
NM_7315	STAT1	-1	-1					1		1		
NM_3199	TCF4	-1	-1	1	1							
NM_18975	TERF2IP	-1	-1					1		1		
NM_3236	TGFA	-1	-1	1							1	
NM_4817	TJP2	-1	-1							1	1	
NM_14683	ULK2	-1	-1		1					1		
NM_376	VDR	-1	-1	1	1							
NM_13245	VPS4A	-1	-1					1		1		
NM_3385	VSNL1	-1	-1	1	1							
NM_14943	ZHX2	-1	-1	1	1							
Total of changes (%)				22%	19%		9%	7%		20%	23%	

Upregulation

		RNA		H3K27-3M			H3K9-3M			DNA 5mC		
Gene	Symbol	EJ-NHU	RT112-NHU	EJ	RT112	NHU	EJ	RT112	NHU	EJ	RT112	NHU
NM_14171	CRIPT	1	1	1	1		1				1	
NM_7358	MTF2	1	1	1				1		1	1	
NM_1613	SAR1B	1	1	1	1					1	1	
NM_2186	ACN9	1	1	1			1				1	
NM_17812	CHCHD3	1	1	1				1			1	
NM_1316	CSE1L	1	1		1					1	1	
NM_2548	DBI	1	1	1	1					1		
NM_16216	DBR1	1	1	1	1						1	
NM_584	DDX39A	1	1					1		1	1	
NM_5675	DGCR6	1	1				1			1	1	
NM_14597	DNTTIP2	1	1					1		1	1	
NM_4147	DRG1	1	1	1	1						1	
NM_1954	FAM35A	1	1		1					1	1	
NM_6547	IGF2BP3	1	1	1	1			1				
NM_17768	LRRC4	1	1					1		1	1	
NM_18353	MIS18BP1	1	1				1	1			1	
NM_4927	MRPL49	1	1		1					1	1	
NM_2117	MRPS12	1	1		1					1	1	
NM_2519	MYO19	1	1		1					1	1	
NM_4546	NDUFB2	1	1		1					1	1	
NM_4146	NDUFB7	1	1		1					1	1	
NM_1823	NUP133	1	1				1			1	1	
NM_18492	PBK	1	1	1			1	1				
NM_2691	POLD1	1	1	1						1	1	
NM_17917	PPP2R3C	1	1					1		1	1	
NM_3129	QTRT1	1	1					1		1	1	
NM_133	RRM1	1	1	1			1	1				
NM_17827	SARS2	1	1		1					1	1	
NM_3983	SLC7A6	1	1				1	1			1	
NM_3258	TK1	1	1		1					1	1	
NM_17646	TRIT1	1	1		1					1	1	
NM_3474	ADAM12	1	1							1	1	
NM_1634	AMD1	1	1							1	1	
NM_1812	ARMC1	1	1							1	1	
NM_18154	ASF1B	1	1	1	1							
NM_24857	ATAD5	1	1		1					1		
NM_71	ATP5I	1	1							1	1	
NM_15878	AZIN1	1	1							1	1	
NM_18963	BRWD1	1	1							1	1	
NM_24631	C11orf61	1	1	1							1	
NM_4894	C14orf2	1	1				1	1				
NM_144597	C15orf4	1	1		1					1		
NM_2377	C1orf163	1	1							1	1	
NM_1884	C2orf24	1	1		1					1		
NM_16647	C8orf55	1	1							1	1	
NM_189	CENPA	1	1	1							1	
NM_1277	CHKA	1	1		1		1					
NM_21615	CHST6	1	1				1	1				
NM_17882	CLN6	1	1							1	1	
NM_14912	CPEB3	1	1	1							1	
NM_6565	CTCF	1	1	1							1	
NM_3472	DEK	1	1							1	1	
NM_19887	DIABLO	1	1	1	1							
NM_14388	DIEXF	1	1				1				1	
NM_2494	DSCC1	1	1							1	1	
NM_18147	FAIM	1	1	1				1				
NM_21922	FANCE	1	1							1	1	
NM_496	FUS	1	1	1	1							
NM_3878	GGH	1	1				1				1	
NM_1224	GTF3C4	1	1	1							1	
NM_1897	HAUS2	1	1	1	1							
NM_4969	IDE	1	1	1							1	
NM_6899	IDH3B	1	1							1	1	
NM_2296	LBR	1	1	1							1	
NM_4526	MCM2	1	1				1				1	
NM_24834	MCMBP	1	1							1	1	
NM_195	MIOS	1	1				1				1	
NM_18944	MIS18A	1	1							1	1	
NM_24629	MLF1IP	1	1		1		1					
NM_4531	MOCS2	1	1					1		1		
NM_15956	MRPL4	1	1	1							1	
NM_3776	MRPL4	1	1	1							1	
NM_15971	MRPS7	1	1							1	1	
NM_2662	MRS2	1	1							1	1	
NM_18133	MSL2	1	1	1							1	
NM_1611	NIP7	1	1		1					1		
NM_4688	NMI	1	1				1				1	
NM_93	NQO1	1	1		1		1					
NM_3297	NR2C1	1	1	1							1	
NM_17615	NSMCE4A	1	1							1	1	
NM_24844	NUP85	1	1					1		1		
NM_16359	NUSAP1	1	1	1							1	
NM_6452	PAICS	1	1	1	1							
NM_2736	PRKAR2B	1	1				1	1				
NM_2789	PSMA4	1	1					1		1		
NM_16395	PTPLAD1	1	1				1				1	
NM_13328	PYCR2	1	1	1	1							
NM_22768	RBM15	1	1							1	1	
NM_2914	RFC2	1	1	1	1							
NM_1837	RHOT1	1	1					1		1		
NM_3683	RRP1	1	1							1	1	
NM_377	RUVBL1	1	1	1							1	
NM_1694	RWDD2B	1	1							1	1	
NM_2967	SAFB	1	1	1							1	
NM_2496	SCD5	1	1				1	1				
NM_3919	SGCE	1	1	1							1	
NM_24745	SHCBP1	1	1				1	1				
NM_3563	SPOP	1	1		1					1		
NM_1598	SRRT	1	1	1	1							
NM_674	SUGT1	1	1	1							1	
NM_3171	SUPV3L1	1	1							1	1	
NM_467	TBCA	1	1							1	1	
NM_4865	TBPL1	1	1							1	1	
NM_321	TFAM	1	1							1	1	
NM_2456	TMEM16C	1	1				1				1	
NM_3876	TMEM11	1	1							1	1	
NM_1451	TMEM14A	1	1					1		1		
NM_17866	TMEM7	1	1							1	1	
NM_17853	TXNL4B	1	1				1				1	
NM_18955	UBB	1	1							1	1	
NM_14777	URB2	1	1	1							1	
NM_161	UTP18	1	1							1	1	
NM_13256	ZNF18	1	1							1	1	
NM_17865	ZNF692	1	1							1	1	
Total of changes (%)				15%	12%		9%	9%		24%	32%	
