# Supplementary material for: Integrated Epigenome Profiling of Repressive Histone Modifications, DNA Methylation and Gene Expression in Normal and Malignant Urothelial Cells
Source: PLoS One. 2012 Mar 7;7(3):e32750. doi: 10.1371/journal.pone.0032750 (PMC3296741; doi:10.1371/journal.pone.0032750)
Supplement: Table S3 — Gene ontology analysis for each epigenetic mark. The table reveals the GO details for all identified pathways associated with each event. (PDF) [file pone.0032750.s010.pdf]

Supplementary table 3. Gene ontology analysis for each epigentic mark.

H3K27-3M only	H3K27-3M and H3K9-3M	H3K27-3M and 5mC	H3K27-3M and H3K9-3M and 5mC	H3K9-3M only	H3K9-3M and 5mC	5mC only
GO:0000280	GO:0001932	GO:0005525	GO:0032559	GO:0007584	GO:0016310	GO:0042542
GO:0007067	GO:0032555	GO:0032561	GO:0030554	GO:0031667	GO:0006793	GO:0000302
GO:0000087	GO:0032553	GO:0019001	GO:0001883	GO:0009991	GO:0006796	GO:0006979
GO:0048285	GO:0017076	GO:0032270	GO:0001882	Serine Thr. pr kinase		GO:0010035
GO:0000279	GO:0042325	GO:0051247	Zinc Finger R C2H2	ID: IPR008271		GO:0070013
Fibronectin, type III	GO:0019220	GO:0009725		ID: IPR017442		GO:0043233
ID: IPR003961	GO:0051174	GO:0009719		Protein Kinase		GO:0031974
ID: SM00060	GO:0045860	GO:0045893		GO:0004674		ID: ribosomal protein
GO:0045892	GO:0033674	GO:0051254		ID: IPR017441		GO:0003735
GO:0051253	GO:0051347	GO:0045941		ID: IPR000719		ID: ribonucleoprotein
GO:0016481	GO:0045859	GO:0010628		GO:0004672		GO:0032268
GO:0045934	GO:0043549	GO:0045935		GO:0006468		GO:0005643
GO:0051172	GO:0051338	GO:0051173		GO:0044427		GO:0046930
GO:0001934		GO:0010557		GO:0005694		GO:0005635
GO:0042327		GO:0031328		GO:0043228		ID: mitochon
GO:0010562		GO:0009891		GO:0043232		GO:0005743
GO:0045937		GO:0055080		GO:0005887		GO:0019866
GO:0031401		GO:0050801		GO:0031226		calcium-binding region:1
GO:0031399		ATP Binding		Extracellular location		domain:EF-hand 2
nucleotide-binding		GO:0005524		ID: transcription		domain:EF-hand 1
GO:0051494		GO:0042981		GO:0045449		GO:0009150
GO:0010639		GO:0043067		ID: DNA binding		GO:0009259
GO:0051493		GO:0010941		GO:0003677		GO:0006164
GO:0051129		ID: IPR015880				GO:0006163
GO:0031497		ID: SM00355				GO:0048660
GO:0034728		ID: IPR013087				GO:0008284
GO:0006333		GO:0030163				GO:0042127
GO:0032103						GO:0034097
GO:0032101						GO:0007243
GO:0048584						GO:0007409
GO:0030335						GO:0048667
GO:0040017						GO:0048812
GO:0051272						GO:0000904
GO:0030334						GO:0048858
GO:0040012						GO:0032990
GO:0051270						GO:0000902
ID: IPR000504						ID: protein transport
ID: IPR012677						GO:0015031
ID: SM00360						GO:0045184
ID: rna-binding						GO:0045944
GO:0003723						GO:0004175
GO:0048545						ID: Protease
GO:0050767						GO:0070011
GO:0051960						GO:0008233
GO:0060284						GO:0044265
GO:0009165						GO:0009057
GO:0034654						GO:0006873
GO:0034404						GO:0055082
GO:0055074						GO:0048878
GO:0055065						
GO:0055066						
repeat:LRR 1						
repeat:LRR 2						
ID: leucine-rich						
GO:0031175						
GO:0048666						
GO:0030030						
GO:0006917						
GO:0012502						
GO:0043065						
GO:0043068						
GO:0010942						
ID: IPR007087						
GO:0051603						
GO:0044257						
