# Supplementary material for: Integrated Epigenome Profiling of Repressive Histone Modifications, DNA Methylation and Gene Expression in Normal and Malignant Urothelial Cells
Source: PLoS One. 2012 Mar 7;7(3):e32750. doi: 10.1371/journal.pone.0032750 (PMC3296741; doi:10.1371/journal.pone.0032750)
Supplement: Table S4 — Phenotype specific gene expression in the epigenetically selected panel as determined using gene expression data [17] . (PDF) [file pone.0032750.s011.pdf]

Supplementary table 4. Phenotype specific gene expression

| Gene ID | Epigenetic Silencing | Epigenetic reexpression | Expression change in UCC | Median expression (fluorescence): median Normal urothelium | median CIS | median NMI | median INV | median all UCC | Difference between UCC and normal | T test difference in expression (p valuse shown): N vs. CIS | N vs NMI | N vs INV | CIS vs. NMI | CIS vs. INV | NMI vs. INV | N vs. UCC |
|---|---|---|---|---|---|---|---|---|---|---|---|---|---|---|---|---|
| NM_004546 | 0 | 1 | -1 | 485.5 | 1029.7 | 1299.0 | 1223.8 | 1110.2 | 624.7 | 0.801 | 0.028 | 0.004 | 0.195 | 0.074 | 0.114 | 0.022 |
| NM_004894 | 0 | 1 | -1 | 508.2 | 741.0 | 952.1 | 1105.3 | 1063.9 | 555.7 | 0.002 | 0.996 | 0.459 | 0.002 | 0.008 | 0.476 | 0.926 |
| NM_007100 | 0 | 1 | -1 | 319.8 | 553.6 | 866.7 | 695.1 | 764.2 | 444.4 | 0.819 | 0.186 | 0.738 | 0.540 | 0.663 | 0.054 | 0.524 |
| NM_018955 | 0 | 1 | -1 | 4055.7 | 4412.7 | 5026.9 | 3865.9 | 4472.2 | 416.5 | 0.049 | 0.702 | 0.087 | 0.088 | 0.439 | 0.187 | 0.166 |
| NM_001316 | 0 | 1 | -1 | 115.5 | 226.6 | 310.1 | 476.2 | 404.2 | 288.8 | 0.513 | 0.132 | 0.020 | 0.077 | 0.029 | 0.040 | 0.122 |
| NM_004607 | 0 | 1 | -1 | 195.5 | 364.7 | 459.4 | 422.6 | 444.5 | 249.1 | 0.055 | 0.005 | 0.692 | 0.001 | 0.027 | 0.014 | 0.226 |
| NM_017866 | 0 | 1 | -1 | 113.0 | 328.4 | 263.9 | 331.7 | 331.8 | 218.8 | 0.108 | 0.003 | 0.040 | 0.011 | 0.017 | 0.123 | 0.059 |
| NM_003472 | 0 | 1 | -1 | 167.3 | 241.3 | 229.7 | 450.7 | 382.0 | 214.7 | 0.411 | 0.015 | 0.000 | 0.352 | 0.019 | 0.010 | 0.001 |
| NM_001316 | 0 | 1 | -1 | 78.4 | 146.4 | 242.7 | 341.6 | 282.9 | 204.5 | 0.235 | 0.024 | 0.071 | 0.001 | 0.003 | 0.475 | 0.054 |
| NM_004927 | 0 | 1 | -1 | 376.7 | 384.2 | 564.2 | 601.4 | 559.7 | 183.0 | 0.986 | 0.071 | 0.173 | 0.154 | 0.196 | 0.614 | 0.054 |
| NM_004546 | 0 | 1 | -1 | 165.3 | 271.9 | 331.6 | 390.8 | 330.0 | 164.7 | 0.639 | 0.001 | 0.000 | 0.007 | 0.000 | 0.079 | 0.000 |
| NM_001033 | 0 | 1 | -1 | 103.0 | 220.9 | 216.5 | 308.3 | 247.8 | 144.8 | 0.473 | 0.002 | 0.000 | 0.007 | 0.000 | 0.279 | 0.000 |
| NM_016647 | 0 | 1 | -1 | 203.0 | 174.9 | 438.8 | 190.8 | 342.8 | 139.8 | 0.009 | 0.062 | 0.000 | 0.868 | 0.009 | 0.158 | 0.001 |
| NM_007100 | 0 | 1 | -1 | 141.7 | 202.5 | 296.8 | 232.8 | 278.1 | 136.5 | 0.005 | 0.005 | 0.115 | 0.001 | 0.001 | 0.148 | 0.224 |
| NM_016359 | 0 | 1 | -1 | 72.7 | 101.9 | 152.3 | 251.3 | 207.7 | 135.0 | 0.226 | 0.120 | 0.002 | 0.678 | 0.627 | 0.135 | 0.005 |
| NM_014597 | 0 | 1 | -1 | 182.5 | 267.2 | 264.9 | 347.6 | 315.9 | 133.4 | 0.022 | 0.648 | 0.000 | 0.029 | 0.445 | 0.000 | 0.005 |
| NM_015878 | 0 | 1 | -1 | 62.8 | 116.4 | 173.3 | 171.4 | 195.7 | 132.9 | 0.353 | 0.341 | 0.095 | 0.809 | 0.010 | 0.015 | 0.854 |
| NM_006565 | 0 | 1 | -1 | 110.7 | 133.4 | 219.5 | 259.1 | 240.8 | 130.1 | 0.569 | 0.002 | 0.001 | 0.047 | 0.034 | 0.354 | 0.005 |
| NM_024844 | 0 | 1 | -1 | 80.1 | 81.8 | 193.6 | 200.2 | 203.0 | 122.9 | 0.363 | 0.023 | 0.973 | 0.216 | 0.366 | 0.026 | 0.110 |
| NM_004147 | 0 | 1 | -1 | 284.4 | 288.7 | 405.7 | 414.6 | 401.9 | 117.5 | 0.040 | 0.107 | 0.001 | 0.355 | 0.111 | 0.019 | 0.000 |
| NM_016001 | 0 | 1 | -1 | 57.6 | 102.1 | 138.6 | 177.7 | 166.4 | 108.8 | 0.392 | 0.116 | 0.013 | 0.046 | 0.019 | 0.174 | 0.099 |
| NM_003776 | 0 | 1 | -1 | 104.9 | 114.9 | 252.3 | 222.1 | 201.7 | 96.8 | 0.002 | 0.544 | 0.238 | 0.911 | 0.770 | 0.784 | 0.168 |
| NM_002296 | 0 | 1 | -1 | 137.4 | 197.8 | 193.0 | 302.1 | 233.5 | 96.1 | 0.166 | 0.215 | 0.358 | 0.970 | 0.193 | 0.194 | 0.804 |
| NM_004688 | 0 | 1 | -1 | 55.3 | 97.6 | 130.6 | 171.3 | 148.6 | 93.3 | 0.069 | 0.116 | 0.463 | 0.755 | 0.116 | 0.151 | 0.721 |
| NM_004894 | 0 | 1 | -1 | 118.3 | 132.1 | 194.2 | 243.2 | 209.9 | 91.6 | 0.961 | 0.000 | 0.000 | 0.015 | 0.013 | 0.835 | 0.000 |
| NM_001316 | 0 | 1 | -1 | 93.4 | 150.9 | 137.9 | 185.3 | 178.4 | 84.9 | 0.178 | 0.174 | 0.000 | 0.644 | 0.038 | 0.000 | 0.007 |
| NM_024629 | 0 | 1 | -1 | 43.2 | 52.9 | 98.3 | 128.7 | 128.1 | 84.9 | 0.069 | 0.860 | 0.797 | 0.005 | 0.025 | 0.529 | 0.735 |
| NM_004526 | 0 | 1 | -1 | 139.3 | 169.0 | 166.5 | 250.8 | 222.2 | 82.9 | 0.310 | 0.010 | 0.005 | 0.032 | 0.015 | 0.272 | 0.012 |
| NM_019005 | 0 | 1 | -1 | 102.5 | 127.1 | 167.6 | 186.8 | 184.8 | 82.4 | 0.022 | 0.000 | 0.000 | 0.080 | 0.052 | 0.801 | 0.001 |
| NM_018230 | 0 | 1 | -1 | 115.7 | 159.0 | 173.7 | 184.4 | 191.7 | 76.0 | 0.108 | 0.112 | 0.023 | 0.018 | 0.703 | 0.000 | 0.628 |
| NM_007358 | 0 | 1 | -1 | 107.5 | 132.5 | 167.7 | 181.8 | 182.4 | 74.8 | 0.140 | 0.025 | 0.015 | 0.029 | 0.024 | 0.606 | 0.114 |
| NM_016395 | 0 | 1 | -1 | 93.3 | 87.8 | 146.8 | 130.2 | 165.3 | 72.0 | 0.837 | 0.004 | 0.001 | 0.005 | 0.002 | 0.973 | 0.000 |
| NM_019005 | 0 | 1 | -1 | 109.4 | 135.8 | 169.4 | 179.4 | 179.3 | 69.9 | 0.579 | 0.000 | 0.432 | 0.001 | 0.492 | 0.150 | 0.984 |
| NM_019054 | 0 | 1 | -1 | 146.7 | 188.1 | 166.8 | 211.9 | 215.9 | 69.2 | 0.418 | 0.001 | 0.012 | 0.014 | 0.076 | 0.447 | 0.000 |
| NM_018307 | 0 | 1 | -1 | 100.9 | 106.6 | 137.1 | 181.5 | 170.1 | 69.2 | 0.037 | 0.001 | 0.002 | 0.000 | 0.000 | 0.258 | 0.002 |
| NM_002967 | 0 | 1 | -1 | 148.8 | 117.0 | 232.4 | 220.9 | 211.5 | 62.7 | 0.168 | 0.001 | 0.001 | 0.000 | 0.000 | 0.247 | 0.000 |
| NM_018120 | 0 | 1 | -1 | 50.9 | 61.6 | 95.7 | 116.8 | 113.4 | 62.5 | 0.036 | 0.019 | 0.001 | 0.964 | 0.115 | 0.116 | 0.000 |
| NM_007358 | 0 | 1 | -1 | 73.2 | 122.6 | 134.4 | 128.0 | 134.5 | 61.3 | 0.372 | 0.000 | 0.426 | 0.116 | 0.499 | 0.001 | 0.453 |
| NM_004146 | 0 | 1 | -1 | 43.1 | 26.2 | 109.8 | 109.1 | 101.2 | 58.1 | 0.084 | 0.180 | 0.521 | 0.001 | 0.006 | 0.287 | 0.521 |
| NM_001033 | 0 | 1 | -1 | 72.7 | 72.5 | 106.8 | 113.2 | 120.3 | 47.6 | 0.057 | 0.001 | 0.005 | 0.000 | 0.000 | 0.959 | 0.004 |
| NM_003707 | 0 | 1 | -1 | 92.9 | 102.1 | 128.2 | 165.3 | 137.9 | 45.0 | 0.116 | 0.477 | 0.039 | 0.389 | 0.239 | 0.115 | 0.102 |
| NM_005675 | 0 | 1 | -1 | 122.2 | 82.4 | 170.1 | 171.7 | 163.8 | 41.7 | 0.737 | 0.952 | 0.015 | 0.777 | 0.067 | 0.018 | 0.082 |
| NM_024056 | 0 | 1 | -1 | 287.8 | 249.4 | 324.3 | 360.5 | 326.9 | 39.1 | 0.327 | 0.128 | 0.083 | 0.871 | 0.768 | 0.856 | 0.075 |
| NM_017615 | 0 | 1 | -1 | 43.8 | 58.1 | 80.3 | 83.4 | 82.1 | 38.3 | 0.644 | 0.130 | 0.000 | 0.499 | 0.009 | 0.000 | 0.004 |
| NM_017917 | 0 | 1 | -1 | 45.7 | 71.6 | 67.7 | 85.2 | 83.4 | 37.7 | 0.960 | 0.270 | 0.020 | 0.550 | 0.135 | 0.195 | 0.031 |
| NM_001634 | 0 | 1 | -1 | 60.5 | 88.9 | 68.4 | 99.1 | 93.4 | 32.9 | 0.404 | 0.343 | 0.114 | 0.737 | 0.821 | 0.310 | 0.162 |
| NM_001634 | 0 | 1 | -1 | 59.6 | 98.3 | 59.9 | 101.0 | 90.5 | 30.9 | 0.372 | 0.001 | 0.000 | 0.035 | 0.001 | 0.030 | 0.000 |
| NM_003563 | 0 | 1 | -1 | 85.1 | 67.6 | 93.9 | 114.9 | 114.7 | 29.5 | 0.310 | 0.050 | 0.104 | 0.000 | 0.001 | 0.517 | 0.142 |
| NM_003171 | 0 | 1 | -1 | 77.6 | 102.1 | 94.5 | 112.5 | 106.7 | 29.1 | 0.189 | 0.390 | 0.154 | 0.029 | 0.032 | 0.308 | 0.265 |
| NM_017768 | 0 | 1 | -1 | 36.8 | 45.5 | 51.6 | 68.0 | 64.3 | 27.4 | 0.037 | 0.067 | 0.016 | 0.785 | 0.094 | 0.150 | 0.001 |
| NM_016103 | 0 | 1 | -1 | 18.6 | 42.3 | 33.1 | 46.0 | 45.9 | 27.2 | 0.039 | 0.001 | 0.000 | 0.000 | 0.000 | 0.941 | 0.000 |
| NM_003563 | 0 | 1 | -1 | 152.5 | 150.4 | 152.7 | 179.0 | 179.6 | 27.1 | 0.612 | 0.001 | 0.000 | 0.001 | 0.001 | 0.317 | 0.000 |
| NM_031209 | 0 | 1 | -1 | 129.1 | 87.4 | 182.9 | 141.8 | 154.7 | 25.6 | 0.306 | 0.899 | 0.729 | 0.389 | 0.374 | 0.856 | 0.694 |
| NM_003876 | 0 | 1 | -1 | 158.9 | 151.3 | 183.9 | 203.2 | 182.7 | 23.8 | 0.243 | 0.071 | 0.009 | 0.212 | 0.019 | 0.143 | 0.002 |
| NM_018147 | 0 | 1 | -1 | 20.7 | 27.9 | 35.7 | 43.0 | 43.4 | 22.7 | 0.940 | 0.016 | 0.025 | 0.043 | 0.063 | 0.674 | 0.034 |
| NM_003878 | 0 | 1 | -1 | 35.0 | 49.4 | 38.8 | 69.1 | 57.1 | 22.2 | 0.832 | 0.014 | 0.003 | 0.064 | 0.026 | 0.413 | 0.012 |
| NM_003201 | 0 | 1 | -1 | 82.3 | 78.4 | 111.0 | 108.2 | 103.1 | 20.8 | 0.167 | 0.308 | 0.009 | 0.452 | 0.334 | 0.010 | 0.060 |
| NM_014388 | 0 | 1 | -1 | 57.2 | 72.2 | 74.9 | 74.8 | 76.9 | 19.7 | 0.891 | 0.032 | 0.063 | 0.048 | 0.070 | 0.705 | 0.075 |
| NM_014388 | 0 | 1 | -1 | 75.7 | 86.0 | 109.9 | 92.7 | 95.2 | 19.5 | 0.328 | 0.002 | 0.000 | 0.011 | 0.000 | 0.006 | 0.000 |
| NM_003201 | 0 | 1 | -1 | 40.1 | 30.7 | 59.8 | 60.5 | 59.1 | 19.0 | 0.807 | 0.003 | 0.000 | 0.058 | 0.028 | 0.314 | 0.003 |
| NM_002914 | 0 | 1 | -1 | 53.3 | 57.1 | 74.2 | 63.7 | 72.1 | 18.8 | 0.451 | 0.006 | 0.000 | 0.001 | 0.000 | 0.160 | 0.002 |
| NM_016359 | 0 | 1 | -1 | 48.0 | 56.4 | 53.7 | 67.3 | 64.8 | 16.8 | 0.281 | 0.000 | 0.000 | 0.000 | 0.000 | 0.586 | 0.001 |
| NM_017865 | 0 | 1 | -1 | 182.1 | 142.1 | 275.1 | 193.2 | 198.6 | 16.4 | 0.222 | 0.146 | 0.749 | 0.054 | 0.192 | 0.474 | 0.576 |
| NM_020662 | 0 | 1 | -1 | 12.4 | 13.9 | 24.6 | 32.5 | 28.8 | 16.4 | 0.005 | 0.001 | 0.009 | 0.000 | 0.000 | 0.825 | 0.004 |
| NM_002967 | 0 | 1 | -1 | 22.2 | 17.6 | 38.5 | 38.0 | 38.2 | 16.0 | 0.519 | 0.005 | 0.003 | 0.002 | 0.002 | 0.763 | 0.008 |
| NM_002691 | 0 | 1 | -1 | 49.7 | 50.3 | 65.4 | 69.9 | 64.7 | 15.1 | 0.674 | 0.003 | 0.002 | 0.003 | 0.001 | 0.475 | 0.005 |
| NM_001809 | 0 | 1 | -1 | 30.6 | 44.3 | 32.7 | 62.1 | 44.4 | 13.8 | 0.351 | 0.001 | 0.010 | 0.047 | 0.166 | 0.229 | 0.005 |
| NM_001277 | 0 | 1 | -1 | 80.5 | 89.1 | 85.1 | 78.3 | 92.1 | 11.5 | 0.441 | 0.003 | 0.016 | 0.082 | 0.208 | 0.420 | 0.007 |
| NM_018133 | 0 | 1 | -1 | 40.8 | 42.4 | 40.6 | 57.3 | 51.7 | 10.9 | 0.385 | 0.012 | 0.007 | 0.009 | 0.007 | 0.590 | 0.010 |
| NM_018492 | 0 | 1 | -1 | 18.4 | 20.2 | 24.2 | 33.8 | 29.1 | 10.6 | 0.047 | 0.001 | 0.001 | 0.084 | 0.095 | 0.821 | 0.000 |
| NM_003258 | 0 | 1 | -1 | 86.3 | 79.6 | 79.5 | 117.7 | 95.9 | 9.7 | 0.723 | 0.086 | 0.018 | 0.121 | 0.026 | 0.167 | 0.048 |
| NM_018944 | 0 | 1 | -1 | 75.4 | 68.8 | 79.8 | 83.6 | 84.3 | 9.0 | 0.843 | 0.132 | 0.657 | 0.110 | 0.726 | 0.295 | 0.883 |
| NM_024631 | 0 | 1 | -1 | 37.5 | 36.3 | 40.5 | 41.8 | 45.1 | 7.6 | 0.232 | 0.000 | 0.330 | 0.012 | 0.599 | 0.086 | 0.854 |
| NM_017615 | 0 | 1 | -1 | 119.2 | 100.0 | 124.4 | 113.1 | 126.6 | 7.3 | 0.129 | 0.592 | 0.519 | 0.086 | 0.897 | 0.418 | 0.578 |
| NM_014388 | 0 | 1 | -1 | 35.7 | 36.2 | 41.0 | 41.2 | 42.0 | 6.3 | 0.046 | 0.065 | 0.045 | 0.465 | 0.612 | 0.301 | 0.020 |
| NM_003297 | 0 | 1 | -1 | 29.4 | 28.7 | 30.8 | 33.0 | 35.3 | 5.9 | 0.704 | 0.009 | 0.061 | 0.017 | 0.096 | 0.768 | 0.016 |
| NM_003201 | 0 | 1 | -1 | 30.4 | 34.3 | 32.4 | 34.3 | 35.7 | 5.2 | 0.789 | 0.856 | 0.181 | 0.560 | 0.112 | 0.153 | 0.412 |
| NM_024094 | 0 | 1 | -1 | 35.2 | 44.5 | 35.1 | 37.7 | 40.1 | 4.8 | 0.193 | 0.005 | 0.000 | 0.335 | 0.010 | 0.001 | 0.000 |
| NM_024834 | 0 | 1 | -1 | 50.8 | 58.7 | 46.2 | 57.3 | 55.4 | 4.6 | 0.540 | 0.012 | 0.547 | 0.078 | 0.341 | 0.059 | 0.186 |
| NM_017882 | 0 | 1 | -1 | 59.8 | 57.8 | 73.0 | 69.3 | 64.2 | 4.4 | 0.693 | 0.000 | 0.000 | 0.007 | 0.007 | 0.782 | 0.000 |
| NM_007358 | 0 | 1 | -1 | 10.6 | 9.2 | 13.0 | 13.5 | 14.4 | 3.8 | 0.211 | 0.001 | 0.002 | 0.014 | 0.012 | 0.604 | 0.000 |
| NM_015878 | 0 | 1 | -1 | 39.6 | 30.1 | 49.5 | 44.9 | 41.8 | 2.3 | 0.006 | 0.070 | 0.629 | 0.006 | 0.002 | 0.104 | 0.170 |
| NM_020186 | 0 | 1 | -1 | 10.2 | 11.1 | 11.2 | 12.2 | 12.0 | 1.8 | 0.285 | 0.006 | 0.000 | 0.027 | 0.002 | 0.771 | 0.000 |
| NM_021107 | 0 | 1 | -1 | 32.8 | 33.6 | 36.2 | 33.4 | 32.9 | 0.1 | 0.245 | 0.306 | 0.080 | 0.678 | 0.688 | 0.180 | 0.156 |
| NM_001759 | 1 | 0 | 1 | 54.8 | 54.4 | 47.3 | 61.3 | 54.3 | -0.4 | 0.602 | 0.044 | 0.000 | 0.067 | 0.000 | 0.048 | 0.002 |
| NM_013231 | 1 | 0 | 1 | 30.1 | 32.6 | 29.3 | 31.3 | 29.4 | -0.6 | 0.343 | 0.070 | 0.011 | 0.206 | 0.047 | 0.604 | 0.002 |
| NM_018102 | 1 | 0 | 1 | 10.8 | 11.3 | 9.9 | 8.6 | 9.9 | -0.9 | 0.208 | 0.058 | 0.287 | 0.004 | 0.031 | 0.386 | 0.133 |
| NM_005328 | 1 | 0 | 1 | 14.4 | 17.6 | 12.8 | 13.1 | 13.2 | -1.2 | 0.830 | 0.002 | 0.000 | 0.006 | 0.002 | 0.402 | 0.002 |
| NM_013437 | 1 | 0 | 1 | 27.4 | 33.7 | 23.7 | 25.9 | 25.7 | -1.7 | 0.265 | 0.002 | 0.537 | 0.001 | 0.218 | 0.007 | 0.007 |
| NM_017434 | 1 | 0 | 1 | 99.5 | 68.9 | 172.5 | 85.9 | 96.3 | -3.2 | 0.015 | 0.008 | 0.263 | 0.001 | 0.086 | 0.002 | 0.132 |
| NM_002851 | 1 | 0 | 1 | 12.1 | 13.2 | 8.5 | 8.6 | 8.6 | -3.5 | 0.180 | 0.130 | 0.000 | 0.034 | 0.300 | 0.001 | 0.530 |
| NM_000585 | 1 | 0 | 1 | 15.1 | 17.6 | 12.3 | 10.3 | 11.6 | -3.6 | 0.690 | 0.042 | 0.066 | 0.035 | 0.042 | 0.834 | 0.067 |
| NM_018094 | 1 | 0 | 1 | 87.1 | 83.3 | 76.6 | 62.1 | 83.1 | -4.0 | 0.090 | 0.509 | 0.739 | 0.011 | 0.090 | 0.855 | 0.812 |
| NM_002318 | 1 | 0 | 1 | 61.3 | 49.6 | 64.3 | 63.5 | 56.8 | -4.5 | 0.283 | 0.235 | 0.004 | 0.735 | 0.176 | 0.002 | 0.044 |
| NM_000585 | 1 | 0 | 1 | 61.5 | 95.3 | 44.5 | 57.2 | 56.2 | -5.3 | 0.013 | 0.002 | 0.617 | 0.000 | 0.004 | 0.003 | 0.029 |
| NM_001400 | 1 | 0 | 1 | 30.6 | 40.7 | 23.3 | 25.1 | 24.4 | -6.2 | 0.242 | 0.045 | 0.001 | 0.916 | 0.409 | 0.023 | 0.010 |
| NM_024628 | 1 | 0 | 1 | 52.0 | 48.9 | 41.5 | 61.2 | 45.5 | -6.5 | 0.106 | 0.015 | 0.154 | 0.001 | 0.010 | 0.289 | 0.057 |
| NM_014683 | 1 | 0 | 1 | 53.0 | 55.8 | 42.8 | 46.0 | 46.4 | -6.6 | 0.207 | 0.214 | 0.003 | 0.874 | 0.040 | 0.010 | 0.035 |
| NM_001999 | 1 | 0 | 1 | 20.0 | 25.5 | 14.5 | 13.1 | 13.1 | -6.9 | 0.468 | 0.119 | 0.381 | 0.458 | 0.774 | 0.739 | 0.110 |
| NM_004572 | 1 | 0 | 1 | 48.4 | 39.4 | 44.6 | 32.2 | 39.2 | -9.2 | 0.993 | 0.165 | 0.215 | 0.194 | 0.228 | 0.843 | 0.180 |
| NM_003199 | 1 | 0 | 1 | 78.0 | 84.2 | 32.3 | 79.4 | 68.4 | -9.5 | 0.953 | 0.055 | 0.003 | 0.044 | 0.002 | 0.415 | 0.016 |
| NM_004265 | 1 | 0 | 1 | 100.1 | 98.2 | 85.1 | 100.9 | 90.4 | -9.7 | 0.824 | 0.935 | 0.012 | 0.694 | 0.008 | 0.004 | 0.189 |
| NM_000153 | 1 | 0 | 1 | 48.5 | 43.2 | 40.2 | 36.4 | 37.8 | -10.7 | 0.959 | 0.070 | 0.013 | 0.053 | 0.011 | 0.153 | 0.022 |
| NM_013231 | 1 | 0 | 1 | 65.1 | 83.4 | 26.6 | 57.5 | 51.3 | -13.8 | 0.794 | 0.080 | 0.028 | 0.420 | 0.142 | 0.219 | 0.038 |
| NM_001999 | 1 | 0 | 1 | 47.1 | 69.8 | 29.3 | 43.2 | 33.0 | -14.0 | 0.886 | 0.007 | 0.001 | 0.016 | 0.002 | 0.170 | 0.003 |
| NM_017434 | 1 | 0 | 1 | 76.6 | 58.7 | 88.7 | 52.0 | 62.6 | -14.0 | 0.431 | 0.031 | 0.002 | 0.074 | 0.005 | 0.458 | 0.001 |
| NM_000177 | 1 | 0 | 1 | 91.0 | 56.7 | 79.8 | 75.2 | 75.7 | -15.3 | 0.761 | 0.114 | 0.042 | 0.213 | 0.192 | 0.744 | 0.033 |
| NM_024833 | 1 | 0 | 1 | 102.7 | 72.3 | 95.5 | 88.5 | 85.7 | -17.0 | 0.894 | 0.125 | 0.834 | 0.203 | 0.756 | 0.014 | 0.416 |
| NM_016559 | 1 | 0 | 1 | 48.7 | 49.9 | 31.0 | 25.5 | 25.6 | -23.1 | 0.814 | 0.015 | 0.002 | 0.004 | 0.001 | 0.153 | 0.010 |
| NM_002616 | 1 | 0 | 1 | 160.5 | 114.0 | 164.5 | 119.1 | 137.0 | -23.5 | 0.899 | 0.008 | 0.002 | 0.002 | 0.001 | 0.247 | 0.005 |
| NM_024042 | 1 | 0 | 1 | 116.1 | 66.2 | 101.5 | 113.3 | 88.8 | -27.3 | 0.374 | 0.734 | 0.091 | 0.456 | 0.863 | 0.045 | 0.271 |
| NM_001759 | 1 | 0 | 1 | 68.5 | 62.2 | 37.5 | 38.4 | 40.4 | -28.1 | 0.519 | 0.164 | 0.109 | 0.251 | 0.126 | 0.923 | 0.134 |
| NM_000487 | 1 | 0 | 1 | 142.8 | 99.0 | 133.8 | 118.7 | 114.2 | -28.6 | 0.754 | 0.011 | 0.002 | 0.023 | 0.005 | 0.442 | 0.002 |
| NM_002202 | 1 | 0 | 1 | 51.4 | 47.9 | 20.3 | 19.2 | 20.7 | -30.7 | 0.169 | 0.848 | 0.410 | 0.107 | 0.425 | 0.388 | 0.496 |
| NM_002616 | 1 | 0 | 1 | 137.4 | 154.8 | 104.3 | 110.4 | 105.9 | -31.5 | 0.007 | 0.756 | 0.429 | 0.000 | 0.003 | 0.526 | 0.899 |
| NM_014683 | 1 | 0 | 1 | 83.4 | 75.9 | 42.7 | 42.7 | 45.7 | -37.6 | 0.171 | 0.054 | 0.013 | 0.014 | 0.004 | 0.106 | 0.008 |
| NM_002318 | 1 | 0 | 1 | 102.5 | 74.4 | 50.4 | 48.8 | 49.2 | -53.3 | 0.195 | 0.917 | 0.813 | 0.108 | 0.220 | 0.863 | 0.711 |
| NM_002589 | 1 | 0 | 1 | 158.8 | 131.2 | 109.0 | 101.3 | 99.8 | -58.9 | 0.558 | 0.455 | 0.237 | 0.702 | 0.031 | 0.046 | 0.949 |
| NM_006517 | 1 | 0 | 1 | 152.0 | 138.1 | 95.5 | 96.7 | 92.7 | -59.4 | 0.437 | 0.003 | 0.001 | 0.118 | 0.039 | 0.423 | 0.000 |
| NM_000785 | 1 | 0 | 1 | 155.6 | 182.6 | 86.1 | 87.4 | 86.7 | -68.9 | 0.960 | 0.423 | 0.303 | 0.475 | 0.340 | 0.740 | 0.279 |
| NM_001759 | 1 | 0 | 1 | 302.6 | 212.5 | 91.3 | 262.4 | 214.2 | -88.5 | 0.012 | 0.364 | 0.031 | 0.075 | 0.259 | 0.257 | 0.063 |
| NM_002084 | 1 | 0 | 1 | 244.0 | 188.8 | 117.5 | 175.6 | 151.4 | -92.6 | 0.152 | 0.725 | 0.189 | 0.058 | 0.883 | 0.068 | 0.476 |
| NM_000177 | 1 | 0 | 1 | 769.2 | 425.1 | 493.9 | 944.4 | 674.8 | -94.5 | 0.847 | 0.000 | 0.000 | 0.006 | 0.001 | 0.075 | 0.000 |
| NM_002084 | 1 | 0 | 1 | 363.3 | 199.8 | 173.9 | 282.4 | 193.9 | -169.4 | 0.046 | 0.005 | 0.221 | 0.000 | 0.007 | 0.034 | 0.024 |
